# Single-cell multi-omics analysis reveals candidate therapeutic drugs and key transcription factor specifically for the mesenchymal subtype of glioblastoma

**DOI:** 10.1186/s13578-024-01332-3

**Published:** 2024-12-20

**Authors:** Yufan Yang, Ziyuan Liu, Yerong Wei, Shuai He, Ancheng Gu, Zhiyong Li, Jianlong Li, Zhongyuan Xu, Bohong Cen

**Affiliations:** 1 Clinical Pharmacy Center, Nanfang Hospital, Southern Medical University, Guangzhou, 510515 Guangdong China; 2 Department of Pharmacy, Zhujiang Hospital, Southern Medical University, Guangzhou, 510282 Guangdong China; 3 National Medical Products Administration Key Laboratory for Research and Evaluation of Drug Metabolism & Guangdong Provincial Key Laboratory of New Drug Screening & Guangdong-Hongkong-Macao Joint Laboratory for New Drug Screening, School of Pharmaceutical Sciences, Southern Medical University, Guangzhou, 510515 Guangdong China; 4 Department of Radiation Oncology, Affiliated Cancer Hospital & Institute of Guangzhou Medical University, Guangzhou, 510095 Guangdong China; 5 Department of Orthopedic Surgery, Nanfang Hospital, Southern Medical University, Guangzhou, 510515 Guangdong China; 6 Department of Neurosurgery, Nanfang Hospital, Southern Medical University, Guangzhou, 510515 Guangdong China

**Keywords:** Single-cell RNA sequencing, SnATAC-seq, Glioblastoma, Mesenchymal, Therapeutic drugs, Transcription factor

## Abstract

The inherent heterogeneity of tumor cells impedes the development of targeted therapies for specific glioblastoma (GBM) subtypes. This study aims to investigate the mesenchymal subtype of GBM to uncover detailed characteristics, potential therapeutic strategies, and improve precision treatment for GBM patients. We integrated single-cell RNA sequencing (scRNA-seq), single-nucleus assay for transposase-accessible chromatin sequencing (snATAC-seq), and bulk RNA sequencing datasets to identify core gene modules, candidate therapeutic drugs, and key transcription factors specific to mesenchymal subtype GBM tumor cells which we validated in vitro and human samples. Our analysis encompassed a heterogeneous single-cell landscape of 55,845 cells from tumor and adjacent normal tissues, focusing on the mesenchymal subtype’s adverse prognosis and its association with hypoxia. We identified a core gene module composed of 38 genes and, through pharmacogenomic analysis, found that Trametinib and Dasatinib exhibit increased effectiveness against mesenchymal subtype GBM cells. Furthermore, by incorporating snATAC-seq data, we delineated a crucial regulatory network and pinpointed the key transcription factor *CEBPG*. Our research has highlighted the strong link between the mesenchymal-like (MES-like) properties of GBM and hypoxia, providing valuable insights into candidate drugs and pivotal targets for precision treatment of the mesenchymal subtype.

**Graphical Abstract:**

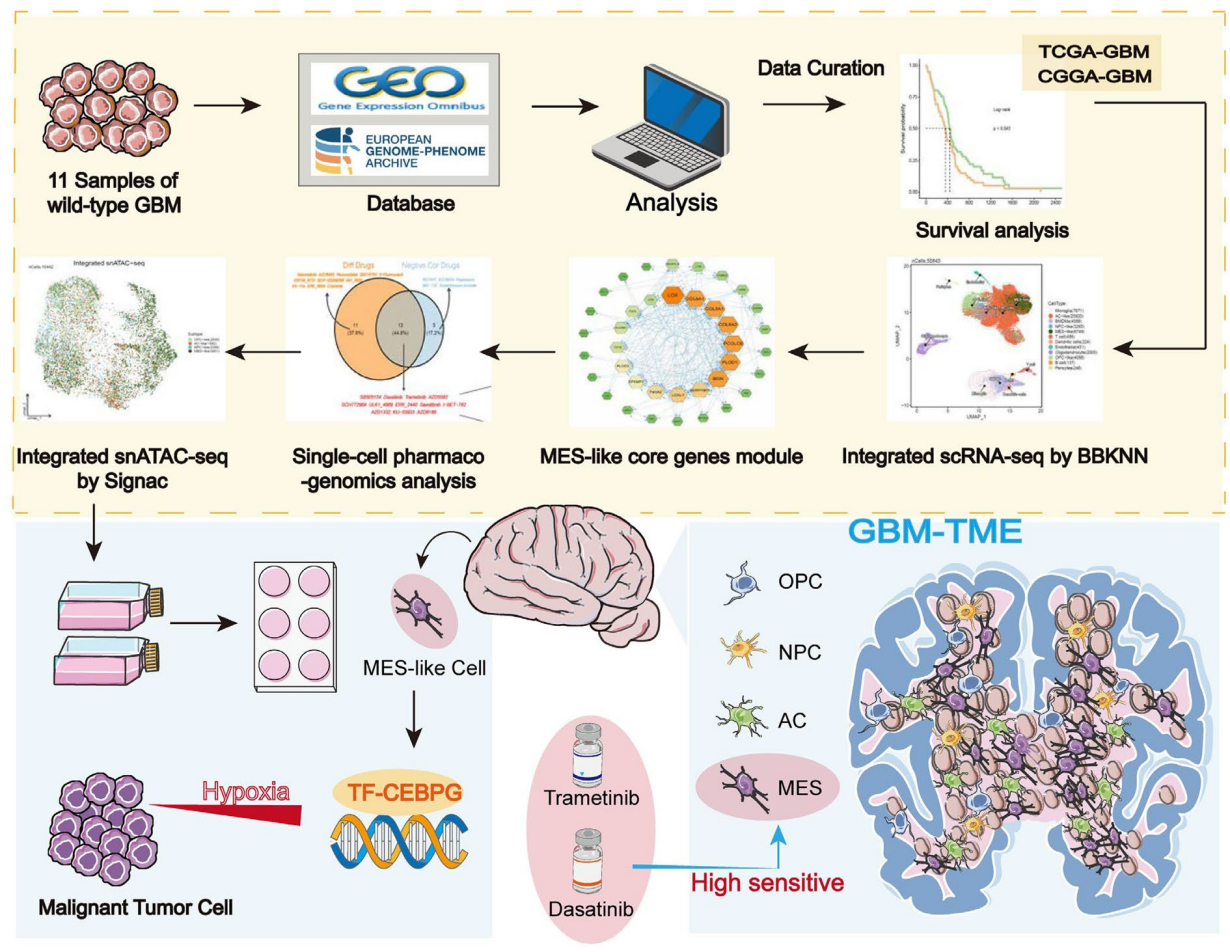

**Supplementary Information:**

The online version contains supplementary material available at 10.1186/s13578-024-01332-3.

## Introduction

Isocitrate dehydrogenase (IDH) - wild type glioblastoma multiforme (GBM) represents an intractable variant of brain tumors [1]. GBM accounts for approximately 57% of all gliomas, making it the most common primary malignant tumor in the adult central nervous system, with a five-year survival rate of just 4.7% [2]. Standard treatments for GBM include surgery, radiotherapy, chemotherapy, and immunotherapy, but their overall effectiveness remains limited [3]. It is characterized by considerable cellular heterogeneity, invasive growth, extensive infiltration into brain tissue, and inevitable recurrence [4]. This high degree of heterogeneity in GBM is attributed to genetic mutations that reshape cellular transdifferentiation and thereby induce tumorigenesis, complemented by the effects of epigenetic programs on crucial phenotypic attributes. Notwithstanding the observed variances in individual GBM cases, concerted efforts have been devoted to discerning prevalent commonalities among patients in order to glean novel treatment strategies [3]. In this age of bulk sequencing, The Cancer Genome Atlas (TCGA) Research Network has crafted a map delineating genomic subtypes of GBM, namely, Classical, Mesenchymal, Neural, and Proneural subtypes, each distinguished by unique features. Intriguingly, within the same tumor in a given patient, different TCGA subtypes can cohabitate in separate or potentially adjacent regions, exhibiting temporal and therapeutic-induced changes [5]. Neftel and colleagues investigated GBM tumor cells using scRNA-seq and discovered that malignant cells share a limited set of cellular states [6]. Some researches indicated that GBM undergoes a proneural-mesenchymal transition (PMT) following radiotherapy, a process that significantly contributes to the high resistance of recurrent tumors to both radiotherapy and chemotherapy [7]. Consequently, recurrent GBM tend to become enriched in the mesenchymal subtype, further acquiring resistance to these treatments. This phenomenon is closely linked to pathological alterations in GBM, which play a critical role in determining clinical prognosis [8]. While these studies have laid the groundwork for understanding the heterogeneity of malignant GBM cells, there is a need for more comprehensive research on the unique characteristics of different cellular states, their influential roles in shaping the tumor immune microenvironment, and their impact on patient prognosis.

Modern medical treatment calls for personalized therapies, and for highly heterogeneous cancers like GBM, research on precision treatment is urgently needed [9]. Currently, it is not difficult to determine the molecular subtype to which a patient belongs, however, there is a scarcity of research on specific treatment approaches for individual subtypes. Moreover, the limitations of temozolomide treatment further underscore the pressing need to discover new drugs for GBM patients [2].

Multi-omics approaches at the single-cell resolution have emerged as a promising method for studying tumor biology [10] [11]. The increasing availability of single-cell RNA sequencing data has uncovered significant features of GBM and its intricate immune microenvironment [12]. Single-cell transcriptomics affords insights into the genetic characteristics of individual cell subtypes, facilitating the identification of targeted treatment options. Traditionally, pharmacosensitivity research has primarily relied on bulk RNA-seq, a method that lacks the granularity required to thoroughly discern inhibitory strategies for each distinct cell type. Moreover, incorporating snATAC-seq allows for a deeper exploration of the epigenetic mechanisms inherent to each subtype [13].

Here, we combined large-scale bulk transcriptome and single-cell transcriptome data to explore the relationship

between the poorest prognosis subtype of mesenchymal (MES) and hypoxia. Through novel computational methods based on high-dimensional gene co-expression analysis, we identified a core gene set in MES-like cells. Using this gene set, we explored emerging therapeutic strategies for patients with the mesenchymal subtype. Firstly, we uncovered potential alternative uses for existing drugs. Secondly, given the increasing recognition of the importance of epigenetics, we further integrated snATAC-seq data to reveal the most specific regulator, CCAAT/enhancer-binding protein gamma (*CEBPG*), for this subtype. We confirmed its tumor-promoting function and its ability to support cell resistance to apoptosis and promote cell invasion under hypoxic conditions through in vitro experiments. Therefore, the use of potential drugs and treatment strategies targeting *CEBPG* provide novel approaches for precise and specific treatment of mesenchymal subtype GBM patients.

## Material and methods

### Data sources

In this study, we obtained single-cell RNA transcription data for wild-type GBM and single-nucleus Assay for Transposase-Accessible Chromatin sequencing (snATAC-seq) data for GBM from multiple sources. The datasets for GSE139448 (scRNA-seq) [14], GSE223063 (scRNA-seq) [15], GSE240822 (snATAC-seq) [16], and EGAC00001002118 (scRNA-seq) [17] were downloaded from the Gene Expression Omnibus (GEO) repository and the European Genome-phenome Archive (EGA). The specific information is in Table S1. The usage of these data sets was duly authorized. The criteria for these samples are as follows: first, they are all wild-type GBM samples; second, their data were obtained using 10x sequencing technology, and the sequencing depths are similar (Fig S6A-B). Additionally, the bulk transcriptomics data of GBM and normal samples were collected from the TCGA database [18], CGGA database [19] and GTEx database [20]. The gene expression data of the cell lines is derived from the Cancer Cell Line Encyclopedia (CCLE) database [21].

### Survival and correlation analysis

In order to explore the role of specific cells in prognosis, we computed subtype scores using the ssGSEA algorithm from the GSVA (v1.48.2) [22]. Subsequently, cox proportional hazards regression models were constructed to identify independent risk factors. Survival analysis was conducted on the TCGA-GBM and CGGA-GBM cohorts through the utilization of Survival (v3.5-5) [23] and Survminer (v0.4.9). The median was selected as the cutoff value to differentiate patients into distinct groups (high or low). The survfit function was employed for the construction of Kaplan-Meier survival curves.

Correlation analysis was conducted to investigate the association between mesenchymal cells and hypoxia. Data analysis and visualization were performed using the R package ggstatsplot (v0.12.0) [24]. p <0.05 was considered as statistically significant. The hypoxia-related gene sets HALLMARK_HYPOXIA and nature_metabolism_hypoxia are derived from previous studies by Arthur Liberzon et al. [25] and Youqiong Ye et al. [26], respectively.

### Single-cell RNA sequencing data process and integration

The Cell Ranger (v3.0.2) pipeline was employed to align FASTQ files to the hg38 10x reference genome (v2.0.0). Preprocessing was conducted using the Scanpy (v1.9.3) package [27]. Firstly, low quality cells were filtered out based on a cutoff threshold of less than 300 total feature RNA and more than 20% mitochondrial RNA and 1% hemoglobin RNA (Fig S6C-D). Doublets were removed using the doubletFinder R package [28]. The gene expression profiles of each cell were utilized for neighborhood graph construction and dimensionality reduction with the UMAP algorithm [29], focusing on the 1500 most highly variable genes. Subsequently, the neighborhood graph underwent batch correction using the BBKNN software [30, 31]. Finally, clustering analysis was performed on this modified neighborhood graph using the Leiden community detection algorithm [32].

### Cell annotations

We translated the h5ad data generated by Scanpy into a Seurat object [33] and identified the malignant cell population using the infercnv (v1.16.0) [34]. The cell types of non-malignant cells were determined using the scGate (v1.4.1) package [35] by analyzing the expression of marker genes. Additionally, we scored the malignant cells based on signatures [36] using the ssgsea function, and assigned the cell type to each cell based on the highest score.

### Trajectory analysis

To explore the dynamic developmental trajectories of cell populations, we conducted trajectory analysis using the CytoTRACE and Monocle2 R packages. CytoTRACE (v0.3.3) [37] facilitated the inference of cellular differentiation orders by quantifying the similarity of gene expression profiles among individual cells. Moreover, Monocle2 (v2.28.0) [38] was utilized to further analyze the trajectory characteristics of distinct malignant subpopulations. These trajectory analyses yielded a comprehensive understanding of the evolving cell states and lineage relationships at the single-cell resolution.

### Identification of hypoxic and normoxic tumor cells

To investigate the relationship between cell subtypes and hypoxic states at the cellular level, we utilized the CHPF Python software [39] for assessing the hypoxic status of tumor cells. This software incorporates seven hypoxia-related gene sets sourced from the Molecular Signature database, all of which are derived from human samples and have their parameters set to default values.

### High-dimensional weighted gene correlation network analysis

The study utilized the hdWGCNA (v0.2.19) package developed by Morabito et al [40, 41] in the implementation of Weighted Gene Co-expression Network Analysis (WGCNA) in single-cell data. This package, specifically designed for analyzing single-cell sequencing data, facilitated the construction of co-expression networks across multiple scales and spatial hierarchies of cells. The WGCNA process started by creating a Seurat object, and the hdWGCNA package employed the k-nearest neighbors (KNN) algorithm to identify similar cell groups for aggregating. The computation of the average or sum expression of these cells resulted in a sparse matrix of metacell gene expression.

The SetDataExpr function was used to specify the MES-like cells for constructing the expression matrix. Subsequently, parameter scans were conducted using the TestSoftPowers function to determine the optimal soft power threshold for constructing the co-expression network. The selection of the soft power threshold, which retained a strong gene-gene correlation adjacency matrix while removing weak connections, was based on evaluating the resulting network topology at different power values. A scale-free topology model was employed, with a minimum soft power threshold set at 0.8 or higher. The ConstructNetwork function was employed to establish the co-expression network using the optimal soft threshold. Furthermore, the ModuleEigengenes function calculated the module eigengenes (ME) by performing principal component analysis (PCA) on a subset of the gene expression matrix specific to each module. The ModuleExprScore function, incorporating the UCell algorithm [42], was utilized to calculate the central gene feature score for each module. Additionally, the FindAllDMEs function was used to assess specific co-expression gene modules in different subtypes of tumor cells. The application of the hdWGCNA package facilitated the identification of robust modules consisting of interconnected genes in single-cell sequencing data, allowing for a comprehensive analysis of WGCNA and exploration of gene co-expression patterns.

### Protein–Protein Interactions and Functional Enrichment Analysis

The hub genes in our screened important module underwent protein–protein interaction analysis in the STRING database [43] and The Cytoscape software was used to identify the Hub genes and for visualization [44]. Functional enrichment analysis was performed using the clusterProfiler (v4.8.1) package [45].

### Tumor immune microenvironment analysis

In this study, we used xCell (v1.1.0) to reveal the correlations between the key mesenchymal signature and immune infiltration [46]. Cell communication pattern was determined using CellChat package [47] by inferring, analyzing, and visualizing the receptor-ligand signaling pathways between highly expressed key modules of tumor cells and other cell types in the tumor microenvironment.

### Drug sensitive analysis

Drug sensitivity scores were obtained from the Genomics of Drug Sensitivity in Cancer (GDSC) database [48] using the oncoPredict (v0.2) [49]. A total of 198 anti-cancer compounds from the GDSC2 dataset were analyzed. In contrast to GDSC1, GDSC2 incorporates more recent sequencing data and experimental results obtained using advanced technologies, equipment, and methodologies, covering research from 2015 onward. To assess drug susceptibility at the single-cell level, we utilized the R package Beyondcell (v1.2.1) [50]. This package allowed us to identify drug sensitivities using scRNA-seq data. Specifically, we employed the drug sensitivity signature collection (SSc) database integrated within Beyondcell. Additionally, we corrected the number of detected genes per cell using recommended guidelines.

### Identification of key regulatory module

Gene regulatory network analysis was conducted using the single-cell regulatory network inference and clustering (SCENIC) approach implemented in pySCENIC (v0.10.0) [51]. The analysis was performed based on the dataset of motifs located within a 20 kb radius around the transcription start site (TSS). The pySCENIC workflow utilized default parameters, and the raw count matrix from all samples was used as the input [52]. Firstly, co-expression modules were calculated, and the weight between transcription factors (TFs) and their target genes was evaluated using GRNBoost2. Then, TFs with direct targets (regulons) were identified using cisTarget. Subsequently, the activity of each regulon in each cell was assessed using AUCell. The function Connection Specificity Index (CSI) was applied to identify regulon modules [53].

### Variance decomposition

To investigate the contribution of TFs in four GBM subtypes, we employed a mixed linear model framework for variance decomposition, with the goal of quantifying how each TF contributes to gene expression variance across subtypes. For each TF, we constructed a mixed linear model, considering the TF’s activity as a fixed effect and the four subtype variables as random effects. These models were fitted using the lmer function from the lme4 R package [54]. The variance components for each TF were subsequently extracted using the VarCorr function. This allowed us to perform variance decomposition for each model and evaluate the extent to which TF activity contributes to differences in GBM subtypes.

### Univariate Cox and Lasso Cox regression.

Using the coxph function from the survival package, univariate Cox regression analysis was conducted on genes in the MES-like module 1, ultimately identifying 38 core genes for subsequent analysis. To precisely identify key genes, the Lasso regression method was applied via the glmnet package [55]. Based on the lambda values corresponding to various numbers of genes, 9 genes were ultimately selected for further analysis of important TF. The screening criterion consistently remained a p-value of less than 0.05.

### Single-nucleus assay for transposase-accessible chromatin sequencing process and analysis

Three samples (GSM7710021, GSM7710022, GSM7710023) were chosen from the GSE240822 dataset [16]. The integration method and filtering criteria employed in this study were consistent with those described in the original research [16]. Gene activity matrices were generated using the GeneActivity function from the Signac (v1.12.0) package [56], and the cell annotation method adhered to the approach outlined in the preceding section.

To evaluate TF-binding accessibility profiles in the snATAC-seq data, We ran chromVAR using wrapper-functions from the Signac package with the default parameters and the JASPAR2022 database. Mapping of the TF motifs to the accessible chromatin regions was performed using the motifmatchr (v1.1.1) R package.

### Cell line culture

Human malignant GBM cell line, U87-MG (ATCC, serial number: HTB-14) and U118-MG (ATCC, serial number: HTB-15). Cells were maintained in DMEM (Gibco, USA) with 10% FBS (Gibco, Shanghai, China) and 100 *µ*g/mL penicillin/streptomycin (Gibco, USA) in a humidified atmosphere containing 5% CO_2_ at 37°C. In hypoxic culture experiments, the gas composition is modified to 1% O_2_, 5% CO_2_, and 94% N_2_. Cells undergo hypoxia for 12 hours before being reverted to standard culture conditions, under which they are cultivated for an additional 48 hours.

### siRNAs construction and transfection

SiRNAs specific to *CEBPG* for silencing its expression (si*CEBPG*-1 to si*CEBPG*-3), including negative control (siNC), were synthesized by RiboBio (Guangzhou, China). Cells (2 × 10^5^ cells/well) were seeded in 6-well culture plate and transiently transfected with 2mL OPTI-MEM medium (Gibco, USA) using siRNA transfection reagent (Genepharma, China).

si*CEBPG*-1 sequences: 5’-GGAACAACAUGGCUGUGAAdTdT-3’ (forward) and 5’-UUCACAGCCAUGUUGUUCCdTdT-3’ (reverse). si*CEBPG*-2 sequences: 5’-GACCAAGGAAUUAAGUGUAdTdT-3’ (forward) and 5’-UACACUUAAUUCCUUGGUCdTdT-3’ (reverse). si*CEBPG*-3 sequences: 5’-GUUCGCCCAUGGAUCGAAAdTdT-3’ (forward) and 5’-UUUCGAUCCAUGGGCGAACdTdT-3’ (reverse).

### Cell viability assay and the 50% inhibitory concentration

U87-MG and U118-MG cells (5×10^3^ cells/well) were seeded into 96-well culture plate with 100*µ*L serum medium and incubated for 24h at 37°C in 5% CO_2_ before performing transfection. After transfection of the cells at 0h, 24h, 48h and 72h, 10*µ*L CCK-8 solution was added to each well, and the OD values of cells at 450nm were detected respectively. Trametinib (GSK1120212, Selleck) and Dasatinib (S1021, Selleck) were dissolved in DMSO to create a 10 mmol/mL stock solution for subsequent experiments. The 50% inhibitory concentrations (IC50) of Trametinib and Dasatinib on U118-MG and U87-MG were determined using the same method as described above. The absorbance at 450 nm was measured at 0 h, 24 h, and 48 h.

### Cell apoptosis assay

After transfection for 48h, cells were collected by trypsin digestion and suspended in 1.5ml EP tubes, PBS wash once (centrifugation at 300g for 5min), then 5*µ*L of Annexin V-FITC and 5*µ*L of the PI staining solution reagent, were added into cells suspension (5 × 10^5^ cells/ml) with 100 *µ*L of 1x Annexin V Binding Buffer of working solution, vortexed and mixed, and incubated for 15 min at room temperature (24°C) and protected from light. Finally, 100*µ*L of 1x Annexin V Binding Buffer was added to each tube and resuspended, and the data were analyzed by flow cytometry with a CytoFLEX flow analyzer (Beckman).

### Cell migration assay

After transfection, cells (2 × 10^5^ cells/well) were seeded into 6-well culture plates containing 2mL serum medium. Parallel lines were drawn at the bottom of the culture plate and detached cells were washed with PBS. Migrated cells at 0h, 24h, 48h and 72h were imaged using ImageJ (v1.8.0) software and the scratch healing rate by migration distance was calculated.

### Cell invasion assay

A double cavity transmission system with an 8*µ*m pore was used in this assay (the upper chamber was pre-added with 60*µ*L matrix solution and incubated at 37°C for 1h). After transfection for 12h, cells (2 × 10^5^ cells/well) were seeded into the upper chamber of the inserts with 200*µ*L serum-free medium, and the lower chamber was filled with 500*µ*L serum medium. After invasion for 48h, inserts were fixed with 100% methanol and then stained with 0.1% crystal violet (Biosharp, China). The invading cells on the inserts were imaged and counted by ImageJ (v1.8.0).

### Colony formation assay

Pre-coat the 6-well plates using a gelatin solution and seed each well with 1 × 10^4^ cells. Allow the cells to adhere before administering transfection and hypoxia treatments. Culture the cells over a 14-day period, re-transfecting on days 4 and 7 to ensure effective suppression of the target TF. On day 14, discard the supernatant and perform three PBS washes. Fix the cells with 4% paraformaldehyde for 15 minutes, then stain with 500 µL of crystal violet for 30 minutes, followed by three PBS washes to remove excess stain. Photograph the cells before employing ImageJ software for cell counting.

### Acquisition of human GBM samples

Human GBM surgical specimens were obtained from Zhujiang Hospital of Southern Medical University (SMU). All human specimens used in this study were approved by the Ethics Committee of SMU (Approval No. 2024-KY-300-01), and informed consent was obtained from the patients or their guardians. Histopathological diagnoses of the GBM specimens were performed by two neuropathologists according to the 2016 World Health Organization (WHO) classification.

### Immunofluorescence staining

To evaluate the expression and localization of *ADM*, *ANGPTL4*, and *CEBPG* in human patient samples, staining was performed using the *ADM* antibody (10778-1-AP, 1:100, Proteintech), *ANGPTL4* antibody (18374-1-AP, 1:500, Proteintech), and *CEBPG* antibody (12997-1-AP, 1:2000, Proteintech) according to the TSA kit protocol. Composite images were acquired using a Nikon confocal microscope (AX) at the designated emission wavelengths and analyzed using ImageJ software.

### Statistical analysis

All statistical analyses and plots were conducted using R (v4.3.1), Python (v3.10.9) and GraphPad Prism (v8.3). The log-rank test was employed for Kaplan-Meier survival analysis, while the Pearson correlation coefficient was used to evaluate linear relationships. A one-way ANOVA was performed for multiple comparisons. P-values were indicated within the plots to denote statistical significance (*P <0.05, **P <0.01, ***P <0.001, ****P <0.0001, ns: nonsignificant).

## Results

### Hypoxia status in GBM samples correlates with risk factor of mesenchymal subtype

**Fig. 1:**
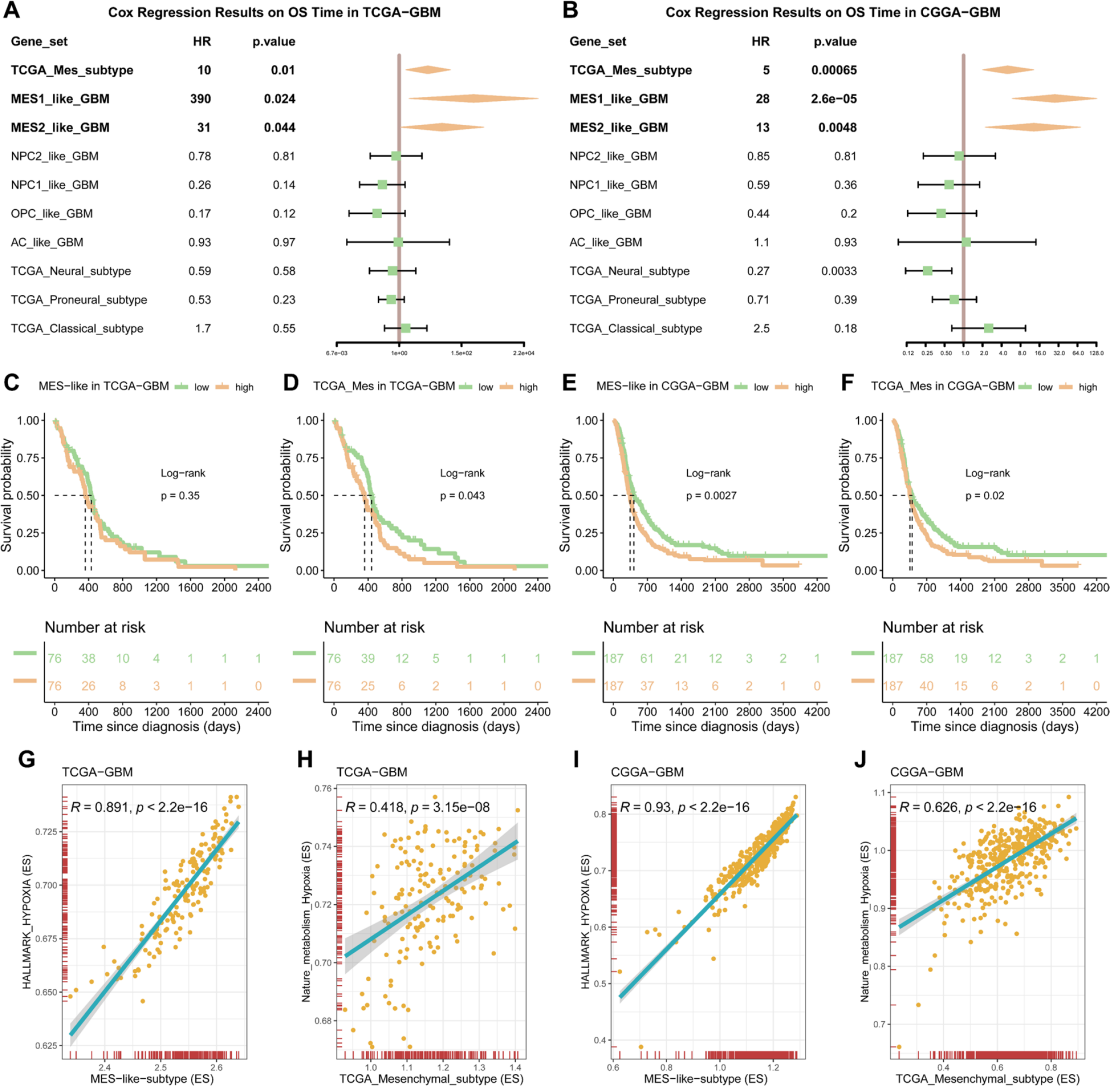
Hypoxic status in GBM correlates with mesenchymal subtype risk factors. **A**-**B** Cox regression results on overall survival (OS) time in the TCGA-GBM and CGGA-GBM, stratified by tumor subtype. The forest plot shows the hazard ratios (HRs) and their 95% confidence intervals (CIs) for each subtype. Subtypes to the right of the dashed line (HR > 1) are associated with a shorter survival time, while those to the left of the line (HR < 1) are associated with a longer survival time. **C**-**F** Kaplan–Meier survival curves for patients stratified by levels of scores of two different MES subtype genesets from previous studies, data derived from the TCGA-GBM and CGGA-GBM cohorts. Based on the median, patients are divided into high-expression and low-expression groups. The number of individuals at risk at each time point is shown below the graph. The p-value from the log-rank test is shown in the figure, indicating the statistical significance of the survival difference between the two groups of patients. **G**-**J** The correlation results of the above MES-related enrichment scores with the HALLMARK_HYPOXIA gene set and Nature_metabolism_hypoxia gene set enrichment scores in the two GBM cohorts respectively

In order to meet the requirements of precision medicine, this study focuses on patients with the most malignant subtype of GBM. The aim is to determine the most malignant subtype of GBM in these patients and understand its characteristics. Initially, gene sets characterizing GBM subtypes identified in two prior studies were utilized to conduct single-sample gene set enrichment analysis (ssGSEA) on samples from the TCGA-GBM samples [5, 6]. The results of the univariate regression analysis showed that regardless of the gene set used, the TCGA_Mes_subtype, MES1_like_GBM, MES2_like_GBM was consistently identified as a high-risk factor with significant implications (Fig 1A). Furthermore, similar results were obtained when integrating data from the CGGA325 and CGGA693 cohorts (Fig 1B). Subsequently, since both the MES1 and MES2 gene sets are associated with poor prognosis, to facilitate the comparison of the four subgroups, the gene sets defining the overall mesenchymal subtype at the cellular level, as described in a previous study [36], was incorporated and used to score the samples. By utilizing the median score to categorize the samples into high and low groups, survival analysis demonstrated a significant association between the mesenchymal subtype and poorer patient prognosis in both TCGA and CCGA cohorts (Fig 1C-F). Therefore, the mesenchymal subtype may be the highest-risk subtype among all subtypes (Fig S1A-F). GBM is characterized by extensive tissue hypoxia which is a key factor in tumor microenvironment that promotes cancer cell spread (invasion) into the healthy tissue to evade this adverse microenvironment. Additionally, phenotypic correlation analysis conducted in the TCGA-GBM and CGGA-GBM cohorts showed significant positive correlations between the mesenchymal subtype and hypoxia [25, 26] (Fig 1G-J), suggesting a clear correlation between the mesenchymal subtype and hypoxia.

### Determining the tumor evolution and hypoxia specificity of MES-like malignant cells

**Fig. 2:**
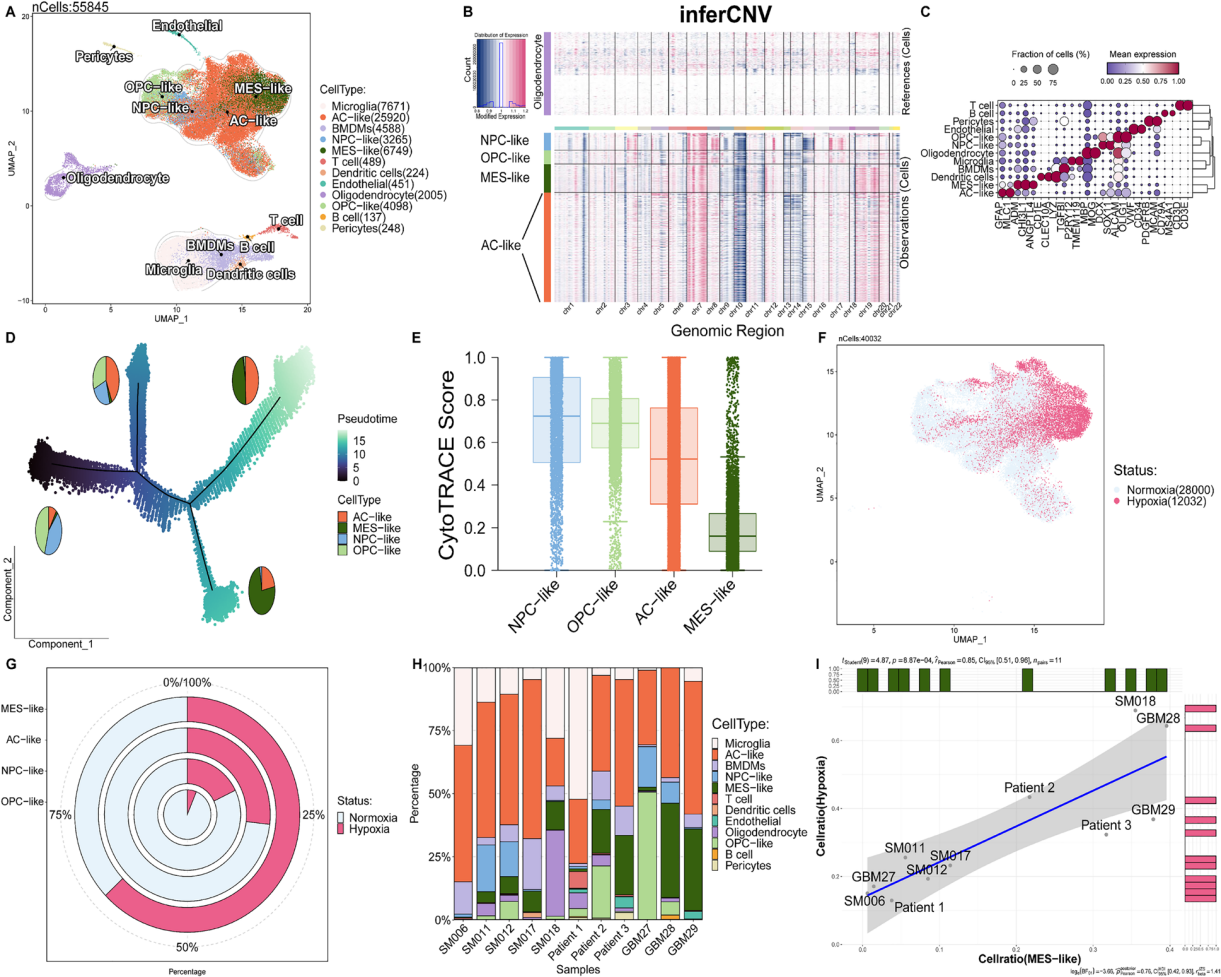
Determining the tumor evolution and hypoxia specificity of MES-like malignant cells. **A** Integration of 11 human GBM scRNA-seq data collected from three individual datasets by BBKNN. A total of 55845 cells were analyzed using UMAP. **B** Inference of copy number variation analysis shows the chromosome 7 gain and chromosome 10 loss in tumor cells compared with normal cells. **C** Dot plot displays the represented markers for each major cell cluster. **D** Pseudotime analysis demonstrates a major transition starting from OPC- and NPC-like to AC- and MES-like cells. Pie charts demonstrate the proportions of each cell type in GBM. **E** Box plot demonstrate the CytoTRACE score of each cell type. **F**-**G** The distribution and proportion of predicted hypoxic cells across different cell types. **H** The distribution of cell populations in each patient is depicted, with cell types color-coded to correspond to those in the UMAP plot. **I** The correlation between the proportion of hypoxic cells and the proportion of MES-like cells in tumor cells from each sample

Bulk transcriptome analysis is insufficient for deciphering the characteristics of different subtypes of tumor cells. Therefore, our objective is to investigate the association between hypoxia and GBM development at the single-cell level via utilizing single-cell data. We conducted rigorous quality control and integrated three prior studies using the BBKNN algorithm (Fig S2A). A total of 55,845 wild-type GBM single-cell transcriptome data were obtained from 11 samples (Fig 2A). To infer malignant tumor cells, we used normal oligodendrocyte cells as a reference and employed infercnv analysis (Fig S2B). Malignant tumor cells were identified through the amplification of chromosome 7 and the loss of chromosome 10 in GBM (Fig 2B). Furthermore, we thoroughly annotated the various cell types (Fig 2C) and observed a significant amplification of chromosome 19 in MES-like cells. Using the CytoTRACE algorithm to

determine the starting point (Fig 2E), trajectory analysis demonstrated a gradual transition in cell proportions from OPC-like and NPC-like cells towards AC-like and MES-like cells as pseudotime progressed (Fig 2D). Notably, the transition of MES-like cells was particularly pronounced, suggesting that MES represents the terminal stage of GBM cell development. Subsequently, using the CHPF tool and seven hypoxia gene sets, we inferred the presence of hypoxic cells (Fig 2F). We found that more than 50% of the cells in the mesenchymal subtype were in a hypoxic state, whereas less than 50% of the cells in other subtypes were hypoxic (Fig 2G). Through correlation analysis, we revealed a strong positive association between the proportion of hypoxic cells (Fig S2D) and MES-like cells in each sample (Fig 2I), despite the observed heterogeneity among samples (Fig 2H) (Fig S2C), but a negative correlation was observed in OPC-like, NPC-like and AC-like cells (Fig S2E-G). This finding suggests that, regardless of the extent of patient heterogeneity, cellular hypoxia status predominantly occurs within the MES subtype cells during GBM progression.

### The identification of a core detrimental gene set in MES-like cells

**Fig. 3:**
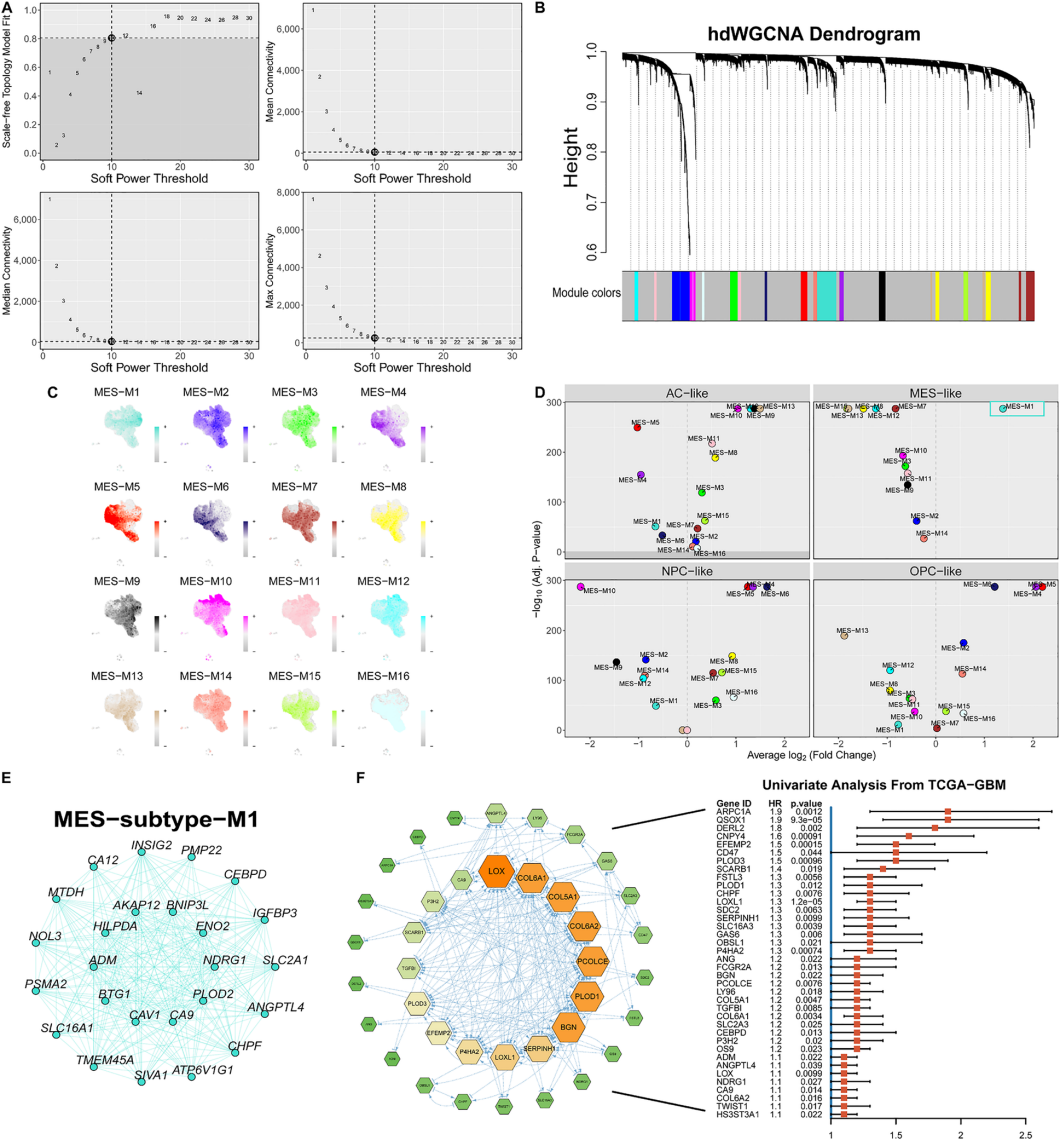
The identification of a core detrimental gene set in MES-like cells. **A** The scale-free topology model was employed to fit the minimum soft power threshold, which was greater than or equal to 0.8, thereby making the constructed network more aligned with the principles of scale-free topology. **B** Utilizing the optimal soft threshold, a co-expression network is constructed whereby genes are categorized into distinct modules, culminating in the creation of a gene dendrogram. The upper section displays the gene hierarchical clustering tree, whereas the lower section comprises the gene modules, also referred to as network modules. **C** Gene scores for each module were computed using the UCell algorithm. **D** Volcano plots of iterative one-versus-all differential module eigengene test results for the four subtypes of tumor cells. **E** The top 25 important genes of Module 1. **F** The left figure represents the gene network diagram obtained after protein–protein interaction and univariate Cox regression screening, while the right figure is the forest plot of the univariate Cox regression of each gene

To elucidate the critical genes that drive MES-like GBM cells, we adopted High-Dimensional Weighted Gene Correlation Network Analysis. This robust method facilitates co-expression network analysis with single-cell resolution and is specifically tailored for scRNA-seq data to identify cellular gene modules and decode the biology of diseases specific to cell types [41]. To explore the core weighted gene co-expression network modules in the mesenchymal subtype cells at the single-cell level, we performed hdWGCNA on the MES-like cells cluster. The TestSoftPowers function was then used to perform parameter scans across various soft power thresholds (ranging from 1 to 30) in a signed network type. With an optimal soft threshold of 10, a co-expression matrix was constructed for the single-cell transcriptome (fraction = 0.05) (Fig 3A). After constructing the network and conducting precise consensus module detection, we ultimately identified 16 expression modules. Genes not included in any module were not considered in subsequent analyses (Fig 3B). The module activities were assessed by UCell, and the results are displayed in Fig 3C. Subsequently, differential analysis was conducted among the modules, revealing that only module 1 exhibited significant specificity in the MES-like cells (Fig 3D). The representative gene network of module 1 is visualized in Fig 3E. To identify poor prognostic genes, all genes within module 1 were subjected to univariate analysis using TCGA-GBM data (Hazard Rate > 1, p-value < 0.05) and further filtered through PPI analysis (Fig 3F). This led to the identification of a detrimental gene module composed of 38 genes, which showed specific and elevated expression in MES-like cells. In descending order of HR, the five most prominent genes are *QSOX1*, *ARPC1A*, *DERL2*, *CNPY4*, and *EFEMP2*.

### The interplay between the M1 module-specific expressed cells and the tumor microenvironment

**Fig. 4:**
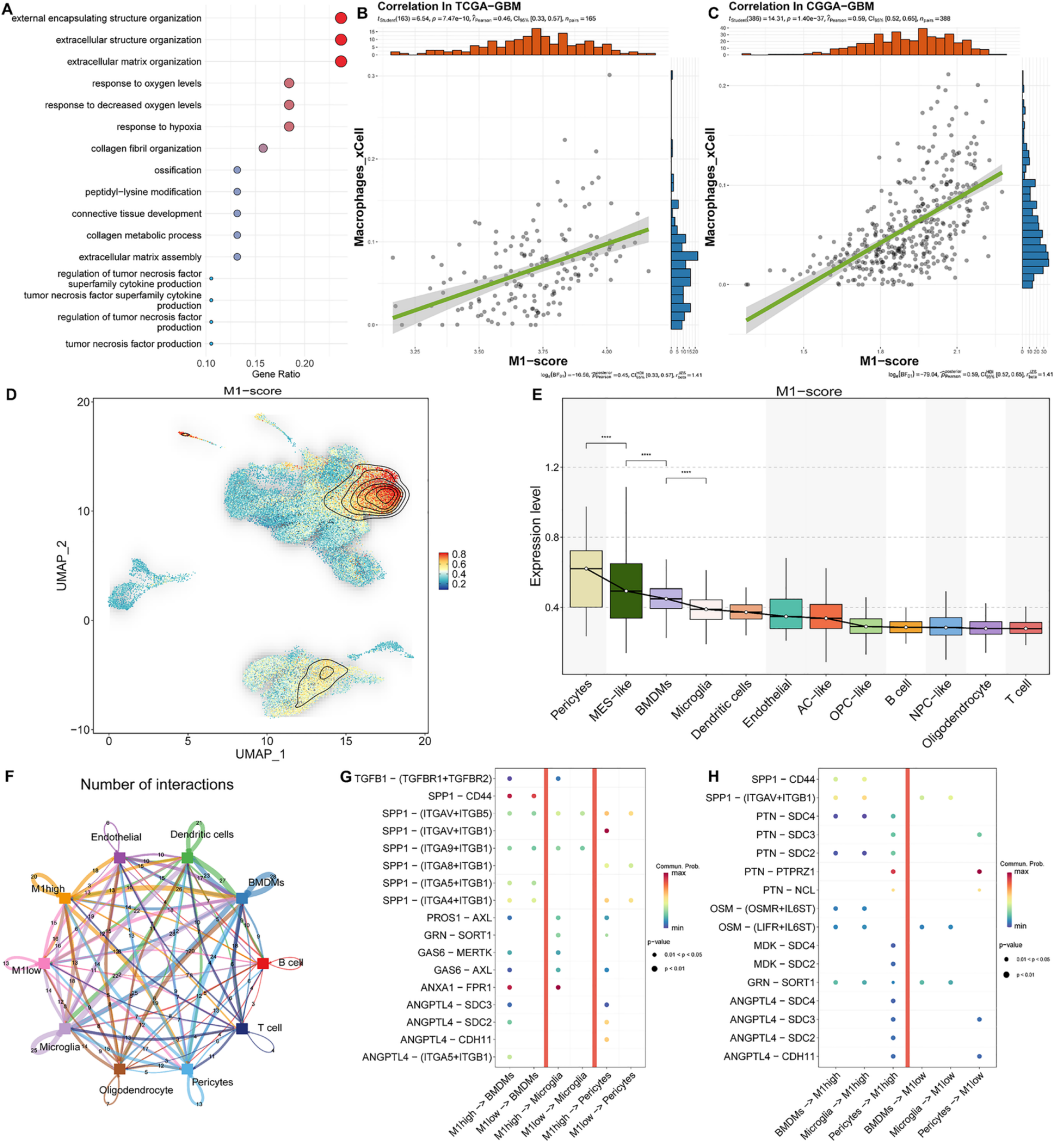
The interplay between the M1 module-specific expressed cells and the tumor microenvironment. **A** The results of the GO enrichment analysis for the 38 core genes of Module 1. **B**-**C** The relationship between the M1-score and Macrophages_xCell. The green line is the fit from a linear regression model, indicating the trend between M1-score and Macrophages_xCell. **D** The density plot of the M1 gene set enrichment score distribution on UMAP. **E** Box plot demonstrate the M1 gene set enrichment score of each cell type. **F** The network of interactions between tumor cells exhibiting high M1 levels and those with low M1 levels in relation to other cellular entities. Node size indicates the frequency of interactions, whereas the thickness of the edges denotes the quantity of significant ligand-receptor interactions observed between the two cellular phenotypes. **G**-**H** The bubble heatmap illustrates the strength of interactions between cells for various ligand-receptor pairs, with the size of each dot reflecting the p-value obtained from the permutation test, and the color of the dot indicating the probability of communication. Areas lacking dots denote a communication probability of zero

In order to investigate the effects of genes in module 1 on the tumor microenvironment, we initially conducted a gene ontology (GO) biological process (BP) enrichment analysis to elucidate their synergistic functions (Fig 4A). The analysis demonstrated a significant enrichment of pathways associated with the extracellular matrix and hypoxia, corroborating our prior results. Moreover, the extracellular matrix plays a crucial role in mediating communication between tumor cells and other cells in the tumor microenvironment [57]. Therefore, we performed immune infiltration analysis using the xCell algorithm on the TCGA-GBM and CGGA-GBM cohorts, and scored each sample using the ssGSEA algorithm (Fig 4B-C). Correlation analysis demonstrated a significant positive association between the scores of module 1 and macrophage infiltration.

At the cellular level, we observed high expression of genes in the module 1 in Pericytes, BMDMs, and MES-like cells (Fig 4D-E). To study their communication effects, we categorized tumor cells into two groups: the M1-high group, which represented the top quartile of M1 scores, and the low group. Cell communication analysis indicated that tumor cell clusters with high expression of the module exhibited stronger communication with the microenvironment (Fig 4F). Furthermore, within these three types of cells with elevated expression of the gene module, we found specific interactions between the M1-high group of tumor cells and microglia, BMDMs cells, and stromal cells through various axis pathways (ANXA1-FPR1, TGFB1-(TGFBR1+TGFBR2), GRN-SORT1, GAS6-MERTK, ANGPTL4-(ITGA5+ITGB1), ANGPTL4-CDH11, and SPP1-(ITGAV+ITGB1)) (Fig 4G). These interactions are believed to contribute to immune suppression in the microenvironment. Additionally, when the tumor group acted as the recipient, we observed a stronger effect of the SPP1-CD44 axis and the PTN-SDC series axis in the M1-high group (Fig 4H). OPN encoded by SPP1 interacts with CD44 and can enhance foci formation, invasion, and tumorigenesis in H-Ras-V12 transformed cells through the Rac-mediated pathway [58]. Pleiotrophin (PTN) expression is upregulated under hypoxic conditions, facilitating cellular migration through its interaction with Syndecan (SDC) [59], suggesting that cells with high expression of the M1 module may overexpress receptors favorable to their development, enhancing hypoxia tolerance and facilitating migration from oxygen-deficient environments through ligand activation.

### Potential MES-like GBM specific therapeutic agents: Trametinib and Dasatinib

Prior research has emphasized the urgent need for more specific drugs targeting the mesenchymal subtype of patients [60]. To find specific drug candidates for MES-like GBM cells, the ridge regression method was employed to identify specific drug candidates for MES-like GBM cells and predict their potential drug response. The training set comprised drug sensitivity data, including IC50 AUC values and CCLE cell line gene expression profiles. Screening criteria were based on the correlation coefficient between the log2FC value relative to M1 low group tumor cells and the tumor score. We randomly divided the cells from the high M1 group and the low M1 group into 50 sets each, with each set considered as a pseudo-bulk sample. The average gene expression level of each cell was taken as the gene expression level of the pseudo-bulk sample. Then, through ridge regression with oncoPredict, we conducted drug sensitivity analysis. The analysis included 198 drugs from GDSC2. Through PCA analysis, it was found that the PCA of high and low groups could be very distinctly separated based on drug sensitivity (Fig S3). This result indicates that there is a significant difference in drug sensitivity between the M1high and M1low cell populations.

**Fig. 5:**
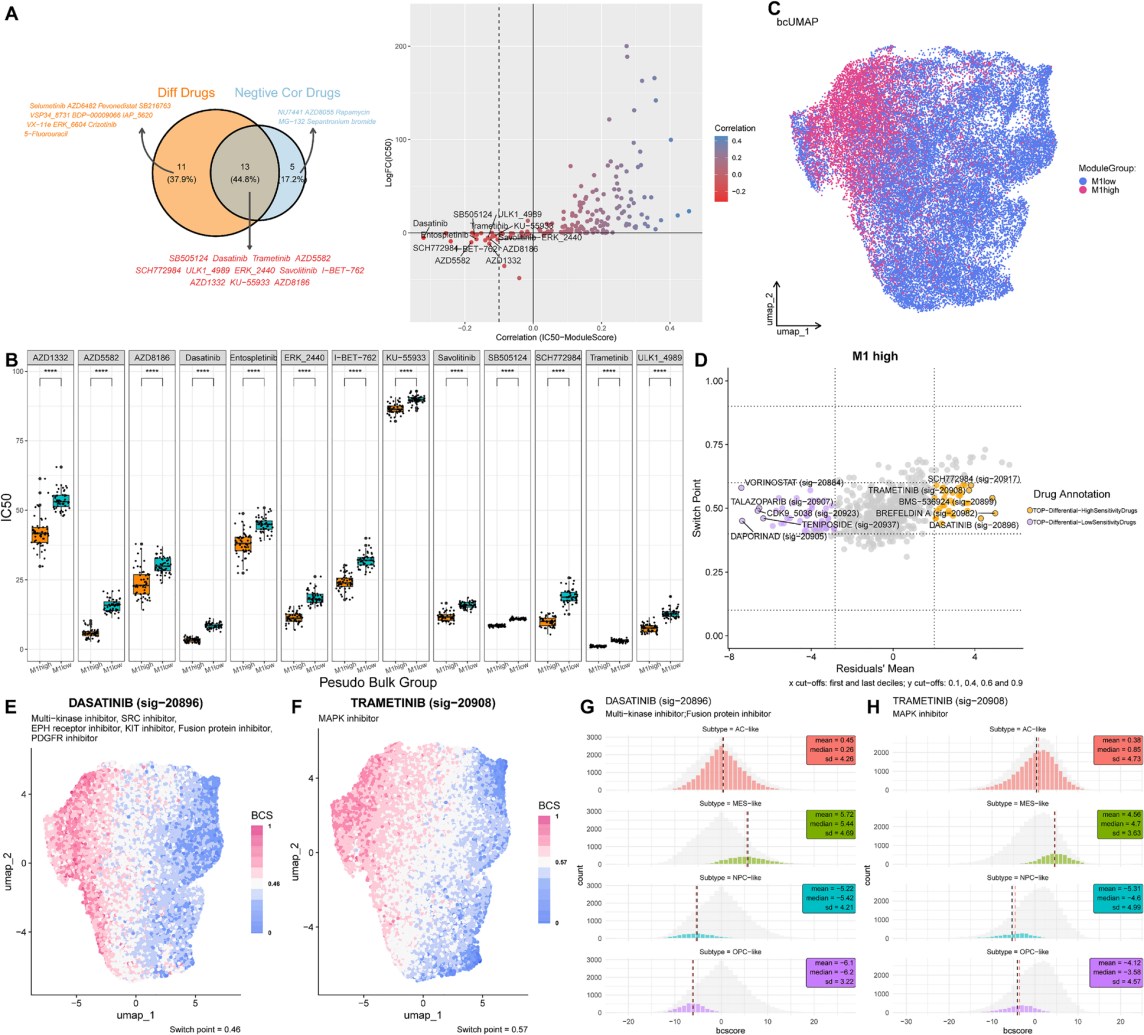
Potential MES-like GBM specific therapeutic agents: Trametinib and Dasatinib. **A** The left graph is a Venn diagram, where the intersection represents drugs that meet both screening criteria; the right graph is a scatter plot, where each point represents a drug, the x-axis represents the correlation between IC50 and M1 scores, and the y-axis represents the logFC from the differential analysis of the pseudo-bulk high and low M1 groups. The dashed line is located at −0.1. **B** Boxplot of IC50 for 13 kinds of drugs in the pseudo-bulk high and low M1 groups. **C** Beyondcell UMAP of tumor cells grouped by high and low M1. **D** The 4 squares plot is a scatter plot of the residual mean and the switch point of the high M1 group cells. The left and right sides respectively display the drugs with low/high sensitivity to selected cells. **E**–**F** Evaluating the drug sensitivity of tumor cells to Dasatinib and Trametinib based on Beyondcell. **G**-**H** Histogram of bc score distribution for 4 subtypes of tumor cells

For a drug to be considered as a potential candidate, it must fulfill two concurrent conditions: an IC50 correlation with the M1 score that is below −0.1 and a negative log2FC (IC50) value [61] (Fig 5A). Ultimately, 12 drugs displaying relatively low IC50 values in the M1 high group and significant impact were selected (Fig 5B).

To evaluate drug sensitivity in malignant cells from both the M1 high and low groups, high-quality cells were retained according to predefined criteria. UMAP embeddings were computed by analyzing transcriptional changes induced by drugs from Beyondcell’s built-in databases, resulting in the identification of clusters similar to those observed based on gene expression (Fig 5C). The top five drugs identified as sensitive to the M1 high group included Dasatinib, Brefeldin A, BMS-536924, Trametinib, and SCH772984 (Fig 5D), with Dasatinib and Trametinib also present in the set of 13 drugs previously predicted. UMAP plots underscored their markedly enhanced sensitivity toward cells within the M1 high group (Fig 5E-F). Bcscore analysis confirmed the sensitivity of both drugs to MES-like GBM cells (Fig 5G-H). The high susceptibility and specificity demonstrated by these two drugs may provide significant insights for treating patients with the MES-like GBM.

### The gene regulatory network specific to the MES-like GBM cells

**Fig. 6:**
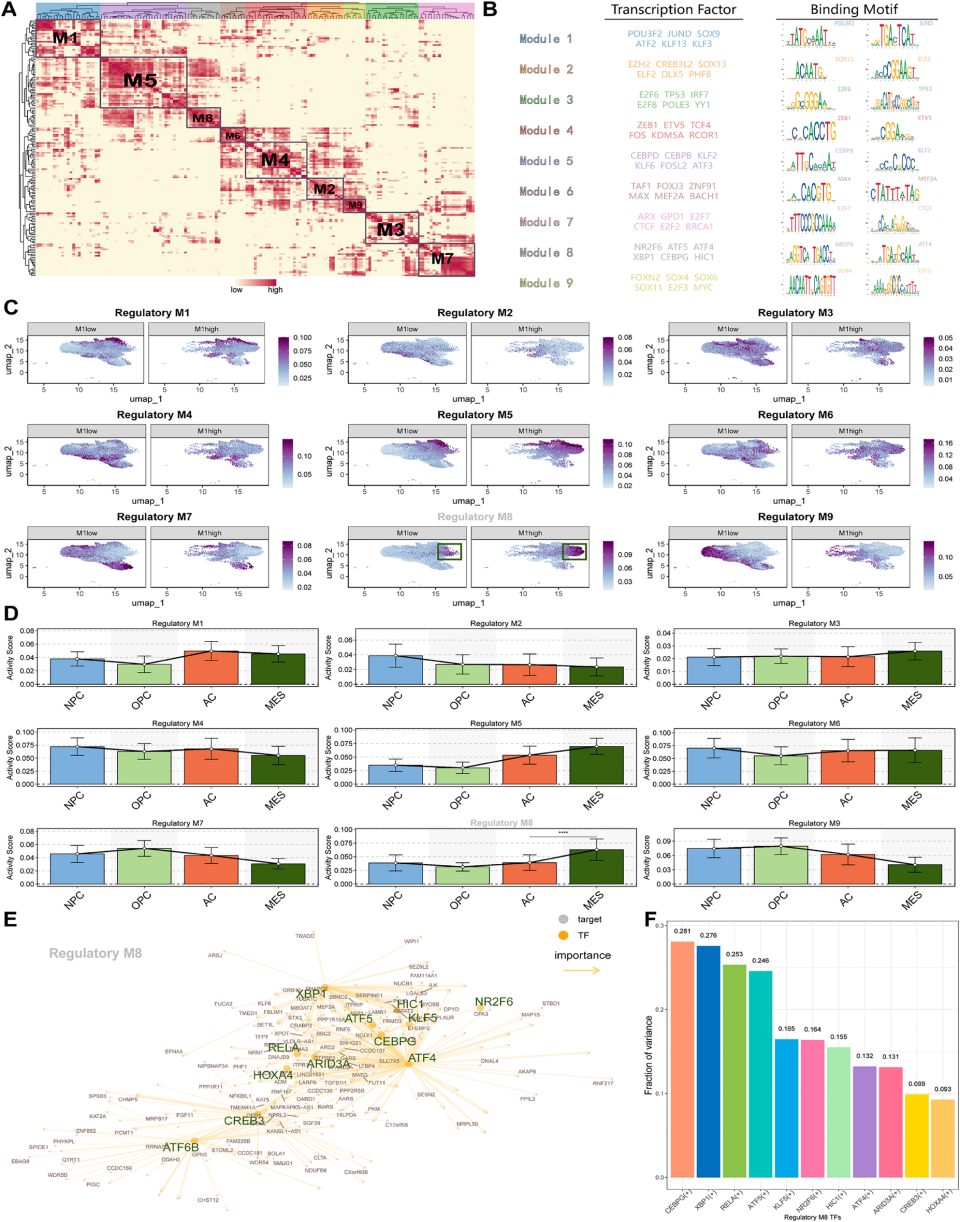
The gene regulatory network specific to the MES-like GBM cells. **A** Distribution of the 9 regulatory modules of transcription factors in malignant cells. **B** Table of six representative transcription factors and their binding motifs for nine regulatory modules. **C** UMAP plot of the activity of nine regulatory modules in the M1 high and low groups. **D** Activity scores of 9 regulatory modules in 4 subtypes of GBM cells. **E** Network diagram of transcription factors and target genes within regulatory module 8. **F** Variance decomposition results of 11 transcription factors in regulatory module 8 across 4 GBM tumor subtypes

Epigenetic regulation can dictate the cellular phenotype and its reprogramming capabilities. Once the critical Gene Regulatory Networks (GRNs) that instigate and preserve cellular state behavior are distinctly identified, it can potentially provide avenues for initiating MES-like GBM reprogramming [62]. To gain insights into the epigenetic characteristics of MES-like cells, we investigated their specific gene regulatory network. SCENIC analysis was performed on malignant tumor cells to calculate the Connection Specificity Index (CSI) (Fig 6A) [53]. Subsequently, clustering was conducted resulting in the classification of the cells into nine regulatory modules(RM), each represented by distinct transcription factors: *POU3F2*, *JUND* (RM1); *SOX13*, *ELF2* (RM2); *E2F6*, *TP53* (RM3); *ZEB1*, *ETV5* (RM4); *CEBPB*, *CEBPD* (RM5); *MAX*, *MEF2A* (RM6); *E2F7*, *CTCF* (RM7); *NR2F6*, *CEBPG* (RM8); *SOX4*,

*MYC* (RM9) (Fig 6B). Notably, *CEBPD* and *CEBPB* did not cluster together with *CEBPG*. Analyzing the previous definition of the M1 high/low group for specific genes, we observed heightened activity in regulatory modules RM5 and RM8 within the M1 high group (Fig 6C). Particularly, RM8 exhibited relatively stronger specific expression in MES-like cells (Fig 6D). Utilizing the results of the SCENIC analysis, a gene regulatory network was depicted for the mesenchymal subtype, representing it through 13 prominent transcription factors (Fig 6E). This implies that the 13 transcription factors are crucial in the gene regulatory network of the mesenchymal subtype, which may facilitate cellular differentiation toward a MES-like phenotype. Lastly, we innovatively employed variance decomposition to dissect the contribution of TFs in RM8 across the four subtypes. The proportion of variance explained by TF activity reflects its influence on subtype variability, where higher values indicate a stronger influence of the TF on subtype, and lower values suggest a weaker impact, revealing *CEBPG*’s significant specificity contribution to the mesenchymal subtype (Fig 6F).

### Essential and specific regulation of MES-like cells by the transcription factor CEBPG

**Fig. 7:**
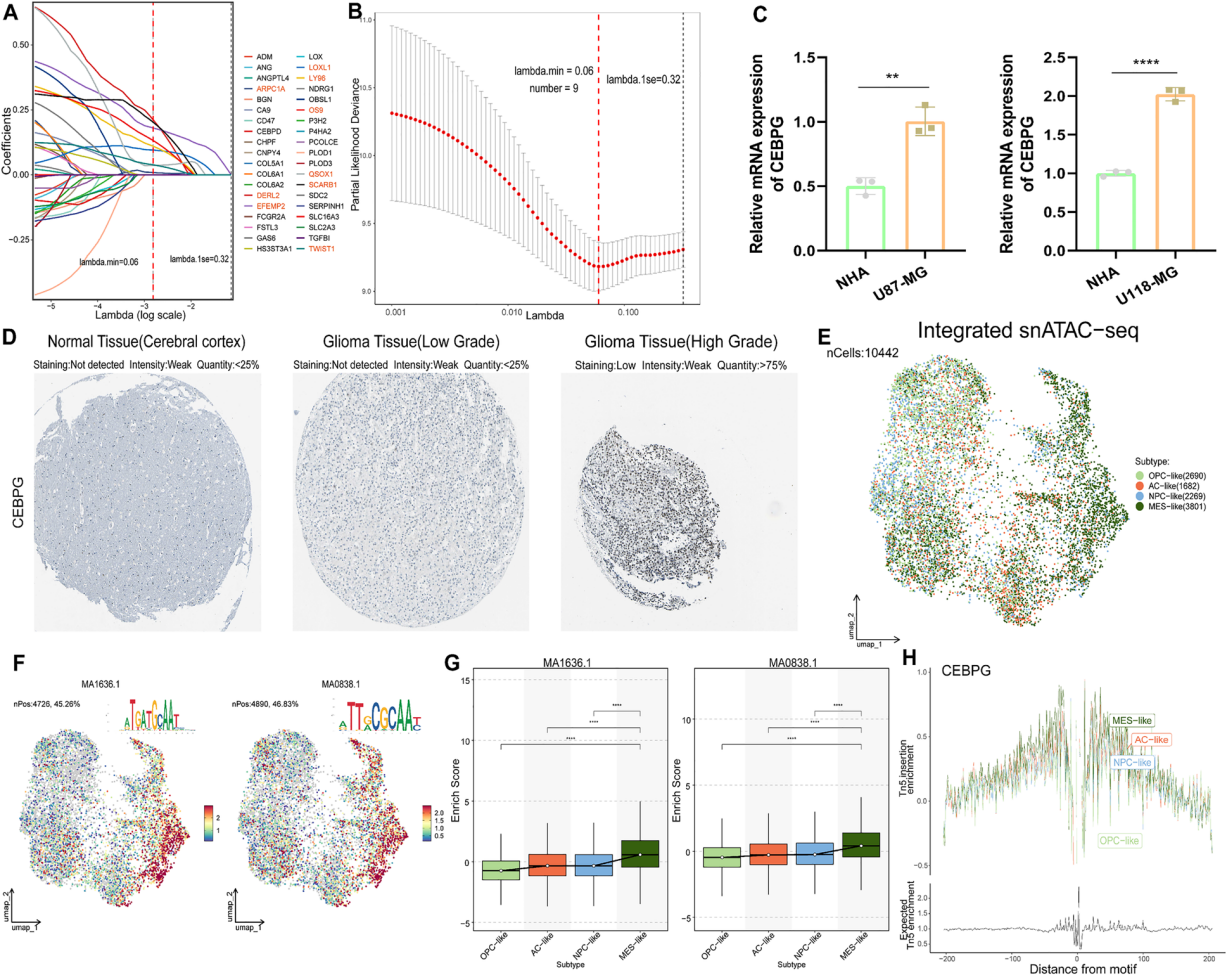
Essential and specific regulation of MES-like cells by the transcription factor *CEBPG*. **A**-**B** Lasso regression analysis was performed to identify 9 M1-related risk genes. **C** The quantitative PCR (qPCR) results for the *CEBPG* gene expression in normal human astrocytes (NHA) and GBM cell lines (U87-MG and U118-MG). **D** Immunohistochemistry results for *CEBPG* in normal cortical tissue, low-grade gliomas, and high-grade gliomas derived from the HPA database. **E** UMAP plot of integrated snATAC-seq (GSE240822) for malignant tumor cells from GBM samples. **F** The functional activity of two binding motifs (MA1636.1 and MA0838.1) of the *CEBPG* transcription factor in GBM cells. **G** Boxplot of enrich score of two binding motifs (MA1636.1 and MA0838.1) of *CEBPG* in 4 GBM subgroups. **H** Genome footprint plot showing the enrichment scores of Tn5 insertions for the *CEBPG* transcription factor in 4 subtypes of GBM cells

To determine the key transcription factor for the mesenchymal subtype, we conducted Lasso regression analysis on genes within the module 1 (Fig 7A). From this module, nine genes were selected for further analysis (Fig 7B). Subsequently, we retrieved TFBS files from the JASPAR database and matched them with the upstream 500bp and downstream 200bp fragments of these genes. Analysis indicates that up to six genes align with the transcription factor *CEBPG*, hinting at its probable regulatory impact on these genes. Notably, it emerges as a pivotal factor in variance decomposition analysis (Fig 6F). *CEBPG*, also referred to as CCAAT/enhancer binding protein gamma, resides on chromosome 19 and is a protein-coding gene implicated in the regulation of gene expression. Given its specificity to the MES subtype, *CEBPG* was chosen for subsequent investigations. Subsequently, we amalgamated data of pan-cancer and matching normal tissues procured from TCGA, GTEx, and CPATC. Comprehensive transcriptomic and proteomic evaluations across a spectrum of cancer types divulged a marked upregulation of *CEBPG*, notably within GBM instances (Fig S4A-B). qRT-PCR analysis revealed that, compared to normal human astrocytes (NHA), *CEBPG* gene expression is significantly upregulated in both U87-MG and U118-MG GBM cell lines (Fig 7C). To validate our findings, immunohistochemistry was performed from the Human Protein Atlas (HPA) dataset (Fig 7D). Integration of three publicly available snATAC-seq datasets (GSE240822) (Fig S4C-D) revealed significant enrichment of two *CEBPG* motifs in MES-like cells (Fig 7E-G), with the tn5 enrichment peak of *CEBPG* being highest in MES-like cells (Fig 7H). Collectively, the comprehensive analysis of multiple omics data highlights the potential importance of *CEBPG* as a crucial and specific transcription factor in the MES-like cells.

### Functional analysis of CEBPG in GBM cell viability, migration, and hypoxic response

Our previous multi-omics analyses have identified *CEBPG* as a pivotal transcription factor in the mesenchymal subtype of GBM. As a result, further investigation was conducted to explore the association between *CEBPG* and tumor development in GBM. For this purpose, signature genes related to hypoxia, Epithelial-Mesenchymal Transition, invasion, metastasis and differentiation were obtained from the CancerSEA database, and the activity scores of each cell subtype were calculated using the ssGSEA algorithm (Fig S5A-E). The results indicated a significant positive correlation between *CEBPG* and these features.

**Fig. 8:**
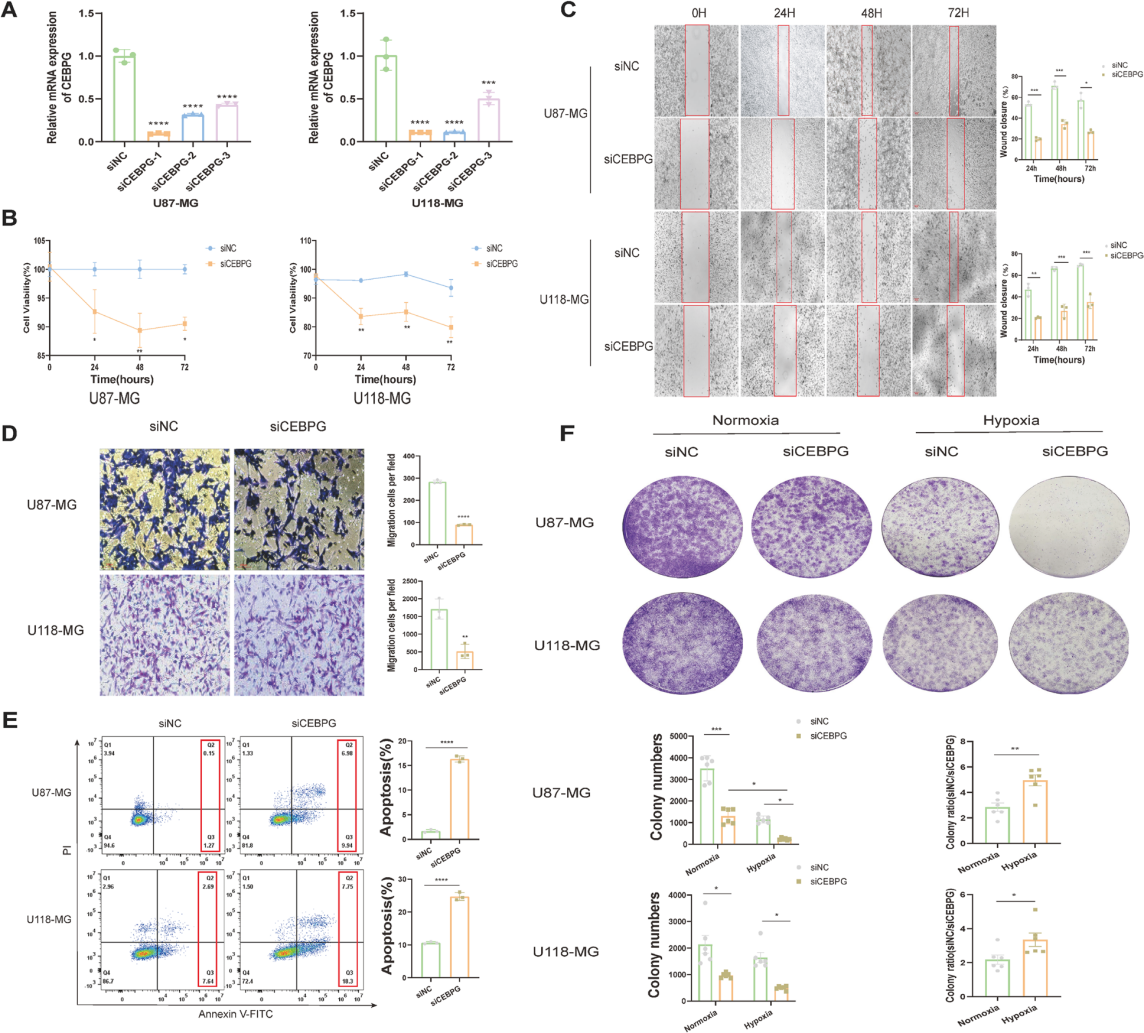
Functional analysis of *CEBPG* in GBM cell viability, migration, and hypoxic response. **A** The knockdown efficiency of si-*CEBPG* in U87-MG and U118-MG tumor cell lines was measured by qRT-PCR experiment. **B** The cell viability of U87-MG and U118-MG after transfection with si-*CEBPG* was measured by CCK-8 assay at 0–72 h. **C** The migration ability of U87-MG and U118-MG cells at 0–72 h after transfection with si-*CEBPG* was assessed by the wound-healing assay. **D** The invasive ability of U87-MG and U118-MG cells after transfection with si-*CEBPG* was examined by transwell invasion assay. **E** 72 h after transfection of U87-MG and U118-MG cells with si-*CEBPG*, cells were harvested and stained with PI and Annexin V-FITC for apoptosis analysis. **F** The tumorigenicity of U87-MG and U118-MG cells after transfection with si-*CEBPG* was evaluated under normoxic and hypoxic conditions by colony formation assay

To evaluate the function of the transcription factor *CEBPG* in GBM cells under normoxic and hypoxic conditions, we first identified cell lines with MES-like characteristics, defined by two MES markers of GBM (*ADM* and *ANGPTL4*), which also serve as independent adverse prognostic indicators (Fig 3F). Analysis of the CCLE database revealed that their expression levels were higher in the U87-MG and U118-MG GBM cell lines, indicating their MES-like properties (Fig S5F-G). This conclusion was subsequently confirmed through qRT-PCR (Fig S5H-I), which showed that the MES characteristics were more pronounced in U118-MG. Finally, we transfected siRNA into human GBM cell lines U87-MG and U118-MG to knock down *CEBPG* expression. The knockdown efficiency was determined using qRT-PCR. We found that si*CEBPG*-1, si*CEBPG*-2, and si*CEBPG*-3 all significantly reduced *CEBPG* expression (Fig 8A). The most efficient, si*CEBPG*-1, was selected for subsequent experiments. To determine the impact of *CEBPG* on cell viability, CCK-8 assays were performed on U87-MG and U118-MG cells post-transfection. Results showed decreased cell viability 24 to 72 hours post-transfection (Fig 8B). To further assess the effect of *CEBPG* on cell migration and invasion, the wound healing and transwell invasion assays were conducted. Results indicated that *CEBPG* knockdown inhibited the migration and invasion capabilities of GBM cells (Fig 8C-D). To further verify *CEBPG*’s role in GBM, flow cytometry and colony formation assays were performed on U87-MG and U118-MG cells post-transient transfection. Flow cytometry analysis showed a significant increase in GBM cell apoptosis ratio after *CEBPG* knockdown (Fig 8E). Colony formation assays demonstrated that cell tumorigenicity was inhibited post-*CEBPG* knockdown, with a more pronounced effect under hypoxic conditions (Fig 8F). This validates our previous findings that *CEBPG* is significantly enriched in hypoxic pathways, enhancing GBM cell resistance to hypoxia and increasing their adaptive capacity in hypoxic tumor conditions.

### Validation of specific expression of CEBPG in MES-like cells in human GBM samples and drug sensitivity

After verifying the specific expression and tumor-promoting functions of *CEBPG* in the MES-like GBM in vitro, we further confirmed these findings in human GBM samples. To this end, we collected GBM patients’ samples and conducted multiplex immunofluorescence analysis (Fig S7A). The results showed a clear co-localization of *CEBPG* with MES subtype markers *ADM* and *ANGPTL4*, further proving the specificity of *CEBPG* in the MES-like GBM. Finally, we assessed the sensitivity of the two previously identified drugs to the MES-like GBM by calculating the IC50 values (Fig S7B). Both drugs were found to significantly reduce the viability of MES-characteristic cell lines U87-MG and U118-MG. Notably, U118-MG cells, which exhibit more pronounced MES features, demonstrated a lower IC50 value, indicating that cells with stronger MES subtype characteristics are more responsive to Dasatinib and Trametinib.

## Discussion

GBM has exhibited substantial heterogeneity in terms of expression profiles and cancer cell plasticity [63] [64]. The heterogeneity, attributed to both genetic and epigenetic variations, continues to pose a significant challenge in GBM treatment [65]. The MES-like state showed significant differences from the cell types detected in the GBM [66], implying its highly malignant nature and the urgent need for new targeted treatment strategies [8]. In light of the critical demand for precision treatments, our research advances prior findings to investigate viable therapeutic approaches for the MES subtype of GBM [5, 6, 36], which is associated with the grimmest prognosis. We have employed high-resolution single-cell technologies to unravel the intricate genetic and epigenetic constitution of the GBM MES subtype.

Through the integration of bulk and single-cell transcriptomic analyses, our study delineates cell state transition patterns predominantly in MES-type cells within the GBM cellular heterogeneity landscape [67]. Hypoxia constitutes a critical therapeutic target across various cancers including GBM. The phenomenon of hypoxia incites a series of molecular reactions, orchestrating a complex network that furnishes cells with the ability to resist therapeutic interventions [68], a characteristic typically associated with MES-like GBM. Despite the extensive data available on the functions of individual genes, the resistance impairment resulting from single-gene inhibition may be offset by compensatory mechanisms within the network. Therefore, the crux of overcoming this challenge lies in the identification of drugs and transcription factors that concurrently regulate multiple genes. Initiating our approach with the exploration of the MES core module, we have unearthed a co-expressed module comprised of 38 genes. This module not only reveals vulnerabilities within MES-like GBM but also furnishes a pragmatic pathway for MES identification. At present, temozolomide remains the sole drug employed in standard therapy regimens. However, temozolomide’s therapeutic outcomes have not met expectations, chiefly due to a lack of tailored therapies for specific subgroups, leading to a scant selection of effective drugs [69]. Past pharmacosensitivity research was largely limited to bulk RNA-seq, which does not adequately address heterogeneity [70]. Consequently, employing single cell pharmacogenomics, we pinpoint two drugs, Dasatinib and Trametinib. Dasatinib is a tyrosine kinase inhibitor (TKI) primarily used in the treatment of certain types of cancer [71]. Meanwhile, dasatinib can cross the blood-brain barrier, which is beneficial for the clearance of MES-like GBM cells [72]. Trametinib, a mitogen-activated protein kinase (MAPK) inhibitor, is primarily utilized in the therapeutic management of melanoma characterized by BRAF V600E or V600K mutations [73]. This agent specifically targets MEK, a critical component of the MAPK signaling cascade, which regulates cellular proliferation and differentiation. Several clinical trials have shown the effectiveness of trametinib in GBM [74, 75]. Our research introduces the novel concept that these two drugs could be exceptionally effective against MES-like GBM. Given their existing FDA approval, they are ideal candidates for drug repurposing. Additionally, MES-like GBM cells display enhanced immunomodulatory effects within the tumor microenvironment, potentially increasing resistance to immune checkpoint blockade (ICB) therapy [76]. Consequently, a combined therapeutic approach involving these two drugs and ICB may represent a promising strategy for future GBM treatment. Extensive research has elucidated the integral role of the hypoxic microenvironment in GBM progression [77], uncovering numerous hypoxia-associated signaling pathways and molecular mechanisms [78] [79]. We employed variance decomposition calculations in conjunction with snATAC-seq technology to match motifs, and discovered *CEBPG*, a transcription factor that is not only malignant but also specifically expressed in the mesenchymal subtype. Our studies indicate that *CEBPG* regulates key MES-specific genes implicated in extracellular matrix (ECM) remodeling and oxygen sensing. We propose that elevated *CEBPG* expression in MES-like GBM is critical for its hypoxia resistance. RM5 and RM8 are in close proximity and may jointly regulate similar biological functions or represent different components of the same signaling pathway. Additionally, *CEBPD*, *CEBPB*, and *CEBPG* belong to the same family and share partially identical structural features [80]. These proteins may act synergistically or complementarily to regulate a shared set of genes, enhancing hypoxia resistance and promoting the formation of MES-like GBM. Interestingly, *CEBPD* and *CEBPB*, despite being from the same family, did not cluster together. Does this suggest that *CEBPG* has a certain degree of delay compared to *CEBPD* and *CEBPB*? Or could their activities be influenced by different factors? Further investigation is warranted. Additionally, previous studies have indicated significant amplification of chromosomes 19/20 in GBM cells from the mesenchymal subtype [81], and *CEBPG* is located on chromosome 19, further supporting our conclusion. This discovery offers a novel viewpoint: *CEBPG* may hold greater importance over *CEBPB* and *CEBPD* within this specific context.

The principal limitation of this study stems from the lack of a stable cell line that accurately represents MES-like GBM cells, impeding our ability to experimentally validate the specificity of drugs efficacy. By employing a variety of standards and methods in our screening processes, we have somewhat mitigated this limitation, thereby bolstering the reliability of our findings. Despite these challenges, our research has yielded novel insights into the precision therapy for the MES-like GBM, characterized by the poorest prognosis.

## Conclusion

In conclusion, we conducted an extensive integration of scRNA-seq on multiple patients diagnosed with wild-type GBM. Within MES-like GBM, we successfully identified a central gene module that is co-expressed. Furthermore, we investigated the correlation between this module and the immune microenvironment, and through pharmacogenomic data mining and validation, we pinpointed two potential precision therapies, Dasatinib and Trametinib, for patients within the mesenchymal subtype. Additionally, we constructed a gene regulatory network specifically tailored to the MES-like cells and, ultimately, through the integration of large-scale snATAC-seq, in vitro validation experiments and human GBM samples, we confirmed the regulatory role of the transcription factor *CEBPG* in the promotion of GBM development and hypoxia resistance. This study provides novel treatment strategies and evidence for the precise treatment of patients with the mesenchymal subtype in the future.

## Supplementary Information

Additional file 1.

## Data Availability

The data used to support the findings of this study are available either online or from the corresponding author upon request.

## Abbreviations

GBM: Glioblastoma
scRNA-seq: Single-cell RNA sequencing
snATAC-seq: Single-nucleus Assay for Transposase-Accessible Chromatin sequencing
UMAP: Uniform manifold approximation and projection
hdWGCNA: High-Dimensional Weighted Gene Correlation Network Analysis
BMDMs: Bone marrow-derived macrophages
DEGs: Differentially expressed genes
MES: Mesenchymal
ES: Enrichment score
TF: Transcription factor
GO: Gene Ontology
logFC: Log fold change
