# Supplementary material for: Single-cell multi-omics analysis reveals candidate therapeutic drugs and key transcription factor specifically for the mesenchymal subtype of glioblastoma
Source: Cell Biosci. 2024 Dec 20;14:151. doi: 10.1186/s13578-024-01332-3 (PMC11662595; doi:10.1186/s13578-024-01332-3)
Supplement: Supplementary file 1 — Additional file 1. [file 13578_2024_1332_MOESM1_ESM.pdf]

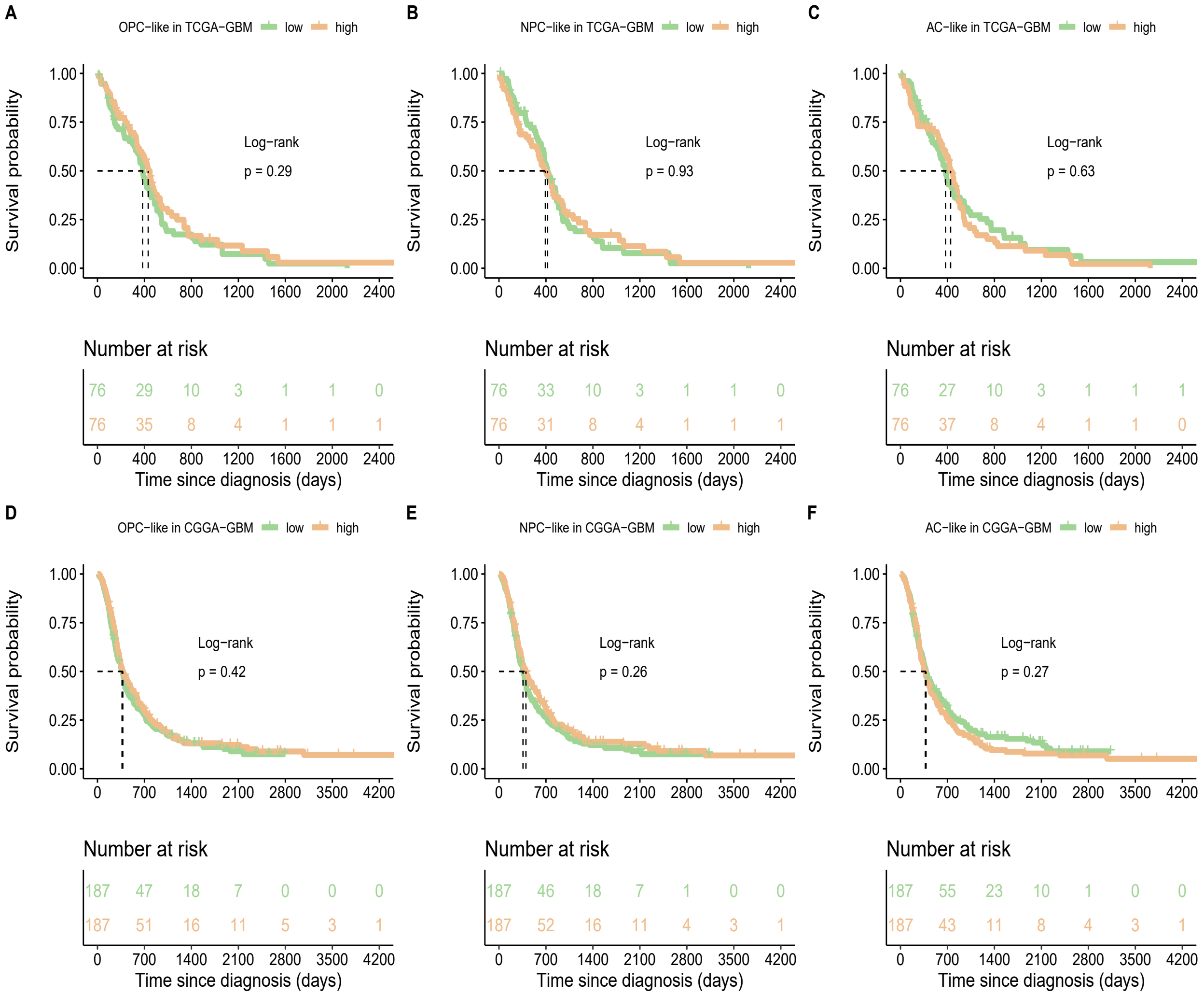

Figure S1: Kaplan-Meier survival curves for patients stratified by OPC-like, NPC-like, and AC-like score levels. (A-F) Survival analysis, utilizing Kaplan-Meier curves, was conducted for patients categorized by OPC-like, NPC-like, and AC-like score levels derived from the TCGA-GBM and CGGA-GBM cohorts. Patients were stratified into high and low expression groups according to the median score.

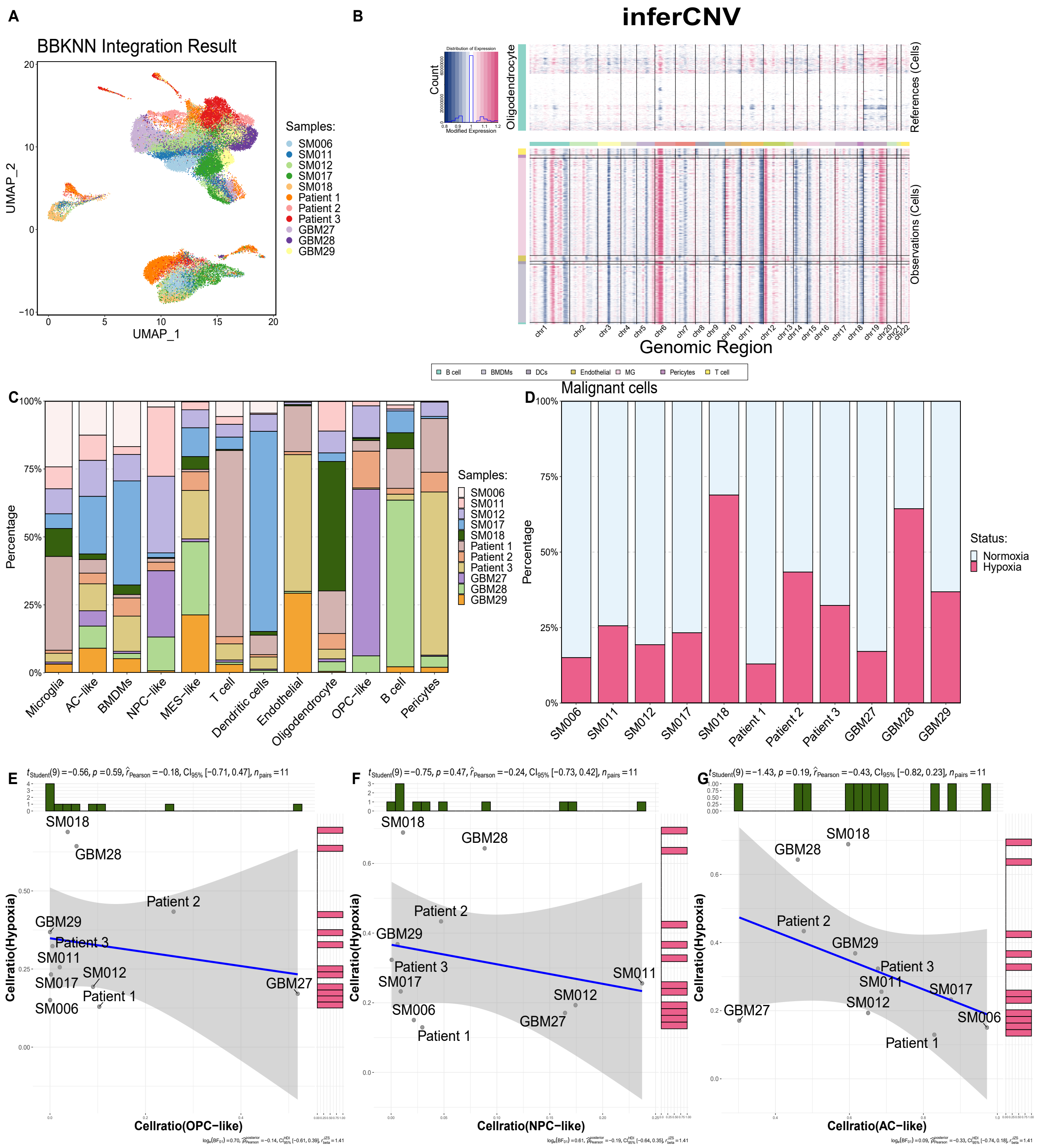

Figure S2: Determining the tumor evolution and hypoxia specificity of MES-like malignant cells by scRNA-seq a nalysis. (A) The UMAP plot presenting the results after BBKNN integration is shown. (B) The inferCNV results for cells other than tumor cells, with oligodendrocytes as a reference. (C) Graph of the proportion of each sample in each type of cell. (D) The proportion of normoxic cells and hypoxic cells in the tumor cells of each sample. (E-G) The correlation between the proportion of hypoxic cells and the proportion of OPC-like, NPC-like and AC-like cells in tumor cells from each sample.

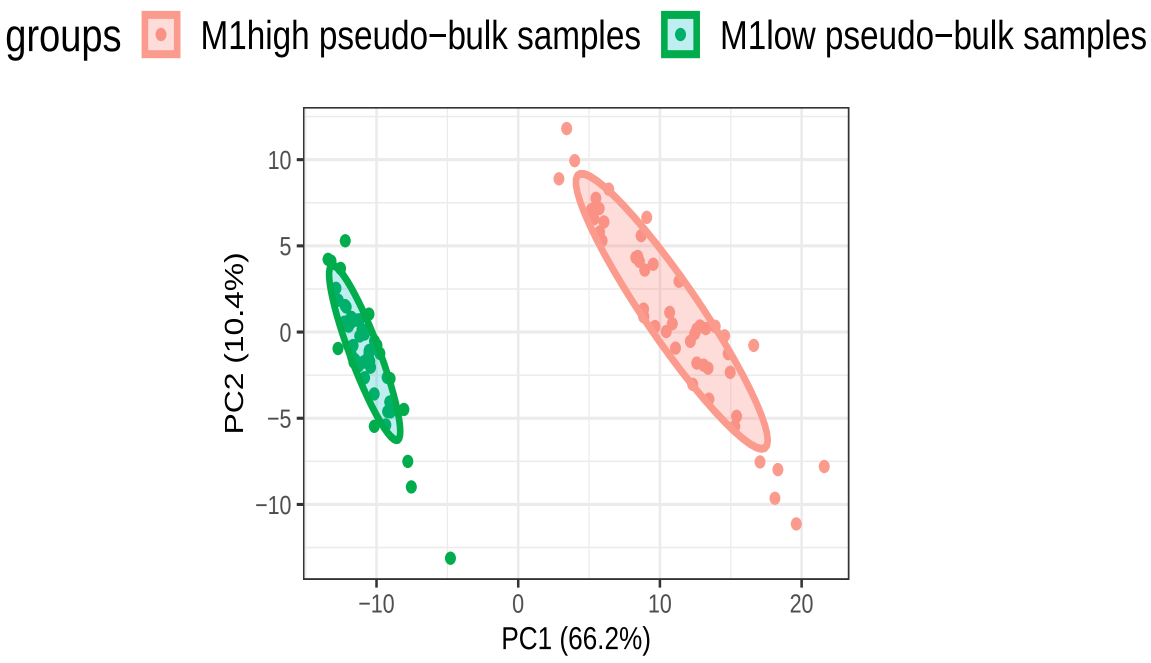

Figure S3: The PCA plot illustrates the drug sensitivity of pseudo-bulk samples characterized as M1high and M1low.

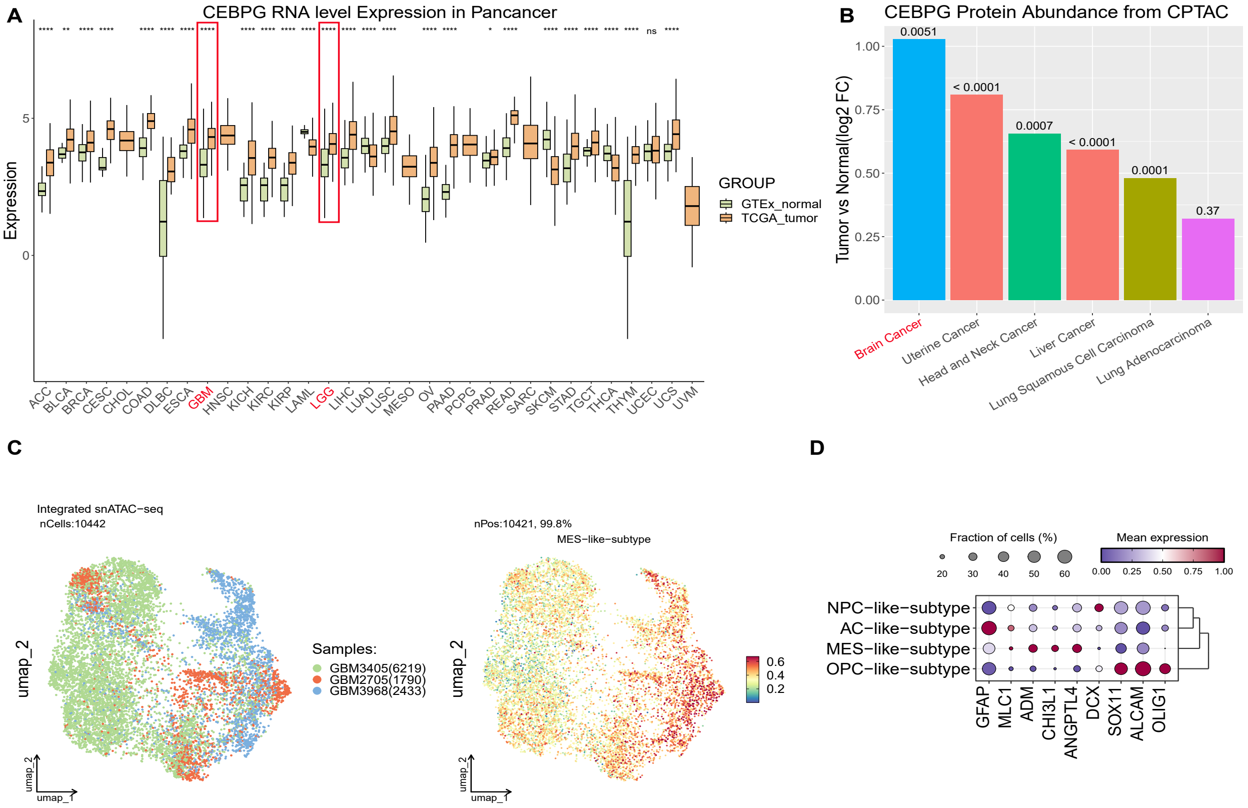

Figure S4: The transcription factor *CEBPG* is a crucial and specific regulator of the MES-like cells. (A) The box plot presents *CEBPG* expression across various cancer types, utilizing data from the TCGA and GTEx databases. (B) The log2 fold-change in the protein abundance of *CEBPG* between normal and tumor tissues, across a range of cancers, was analyzed using data obtained from the Clinical Proteomic Tumor Analysis Consortium (CPTAC). (C) UMAP plot of the integrated tumor cell snATAC-seq data and MES sample scores. (D) Dot plot displays the represented markers for each major GBM cell subclusters.

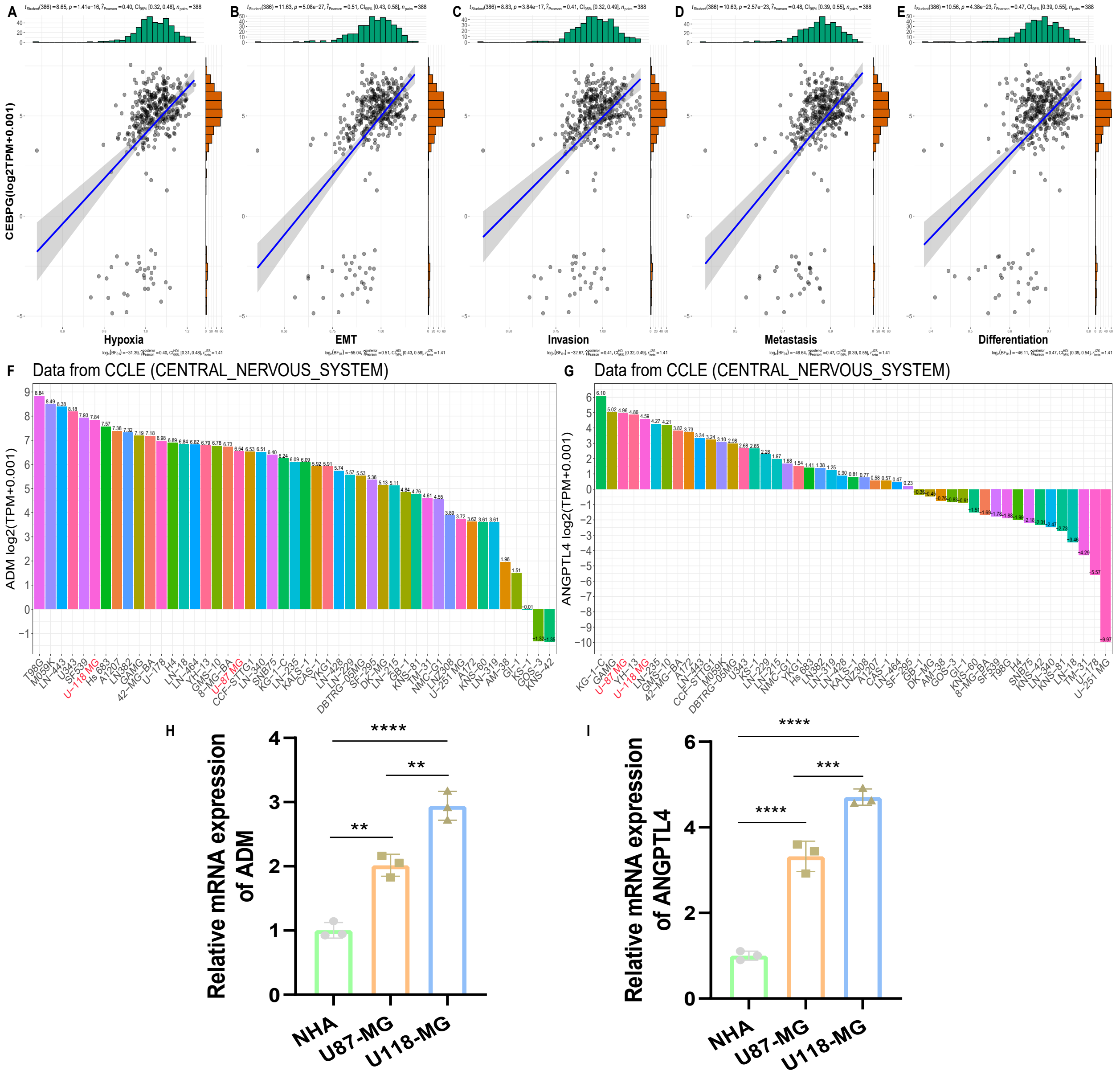

Figure S5: The transcription factor *CEBPG* is linked to the malignancy of GBM cells and supports resistance to hypoxia. The relevance graph demonstrates that *CEBPG* is associated with hypoxia (A), EMT (Epithelial-Mesenchymal Transition) (B), invasion (C), metastasis (D)and differentiation (E) in GBM. (F-G) The bar chart shows the expression levels of MES-like GBM markers (*ADM* and *ANGPTL4*) in cell lines (including U87-MG and U118-MG) related to the central nervous system. (H-I) The qRT-PCR results for the gene expression levels of MES-like GBM markers (*ADM* and *ANGPTL4*) in normal human astrocytes (NHA) and GBM cell lines (U87-MG and U118-MG).

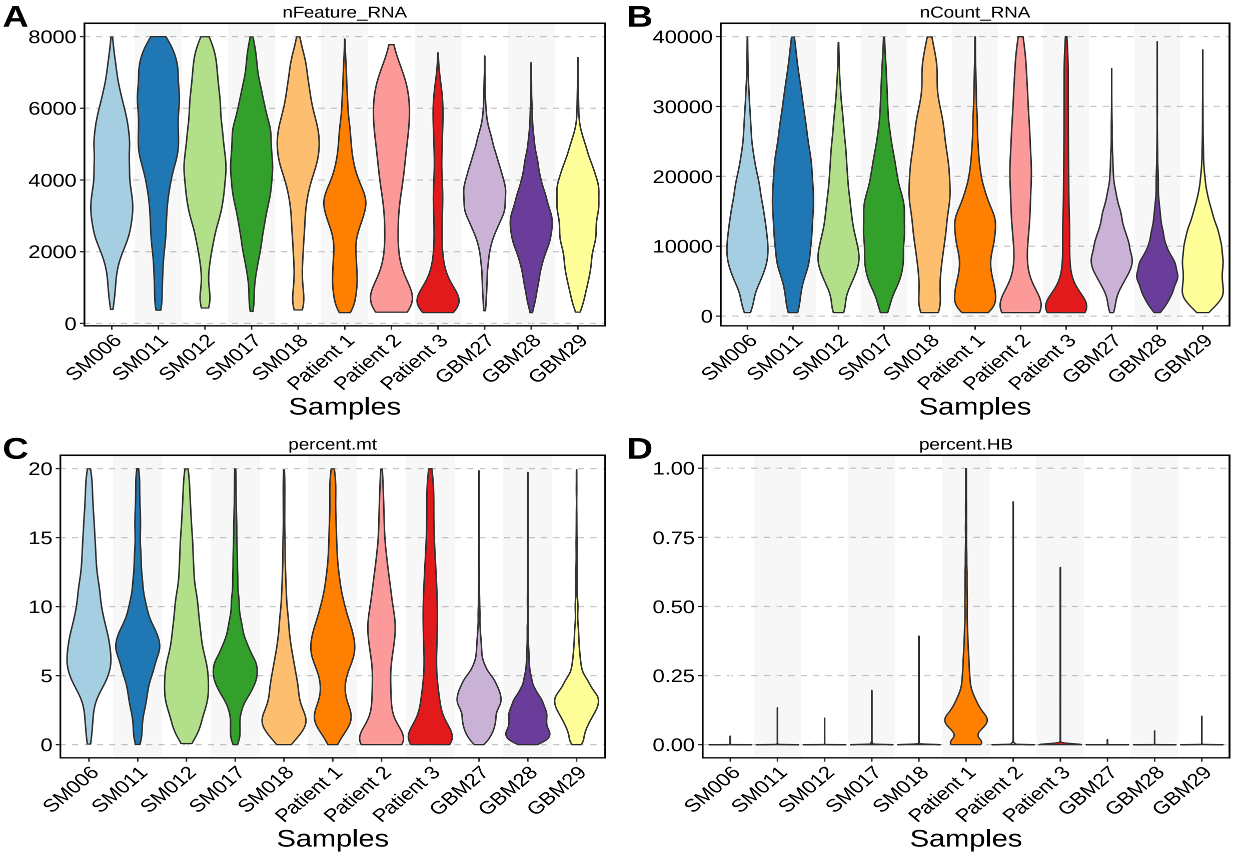

Figure S6: Quality control of single-cell RNA-seq. Violin plots show the number of genes (A), total count values (B), mitochondrial ratio (C), and hemoglobin ratio (D) in each cell of each sample after single-cell data quality control.

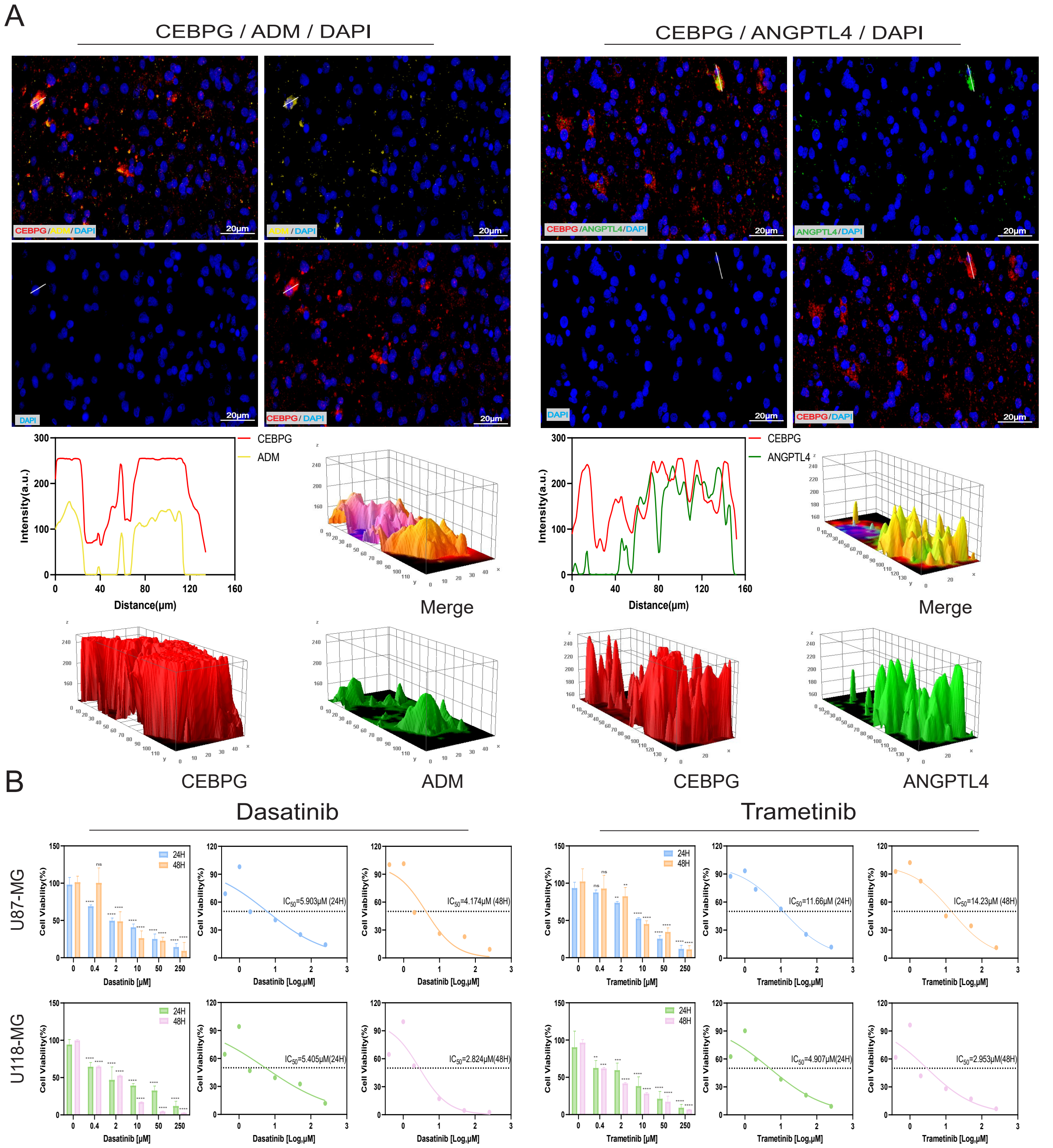

Figure S7: (A) The expression of *ADM* and *ANGPTL4*, markers of the MES-like GBM, is presented alongside *CEBPG* expression and corresponding fluorescence intensity in samples from human GBM patients. (B) The cell viability and IC50 values of Dasatinib and Trametinib were measured in U87-MG and U118-MG cell lines at varying concentrations over 24 and 48 hours.
